# IL-23p19 and CD5 antigen-like form a possible novel heterodimeric cytokine and contribute to experimental autoimmune encephalomyelitis development

**DOI:** 10.1038/s41598-021-84624-9

**Published:** 2021-03-04

**Authors:** Hideaki Hasegawa, Izuru Mizoguchi, Naoko Orii, Shinya Inoue, Yasuhiro Katahira, Toshihiko Yoneto, Mingli Xu, Toru Miyazaki, Takayuki Yoshimoto

**Affiliations:** 1 Department of Immunoregulation, Institute of Medical Science, Tokyo Medical University, 6-1-1 Shinjuku, Shinjuku-ku, Tokyo, 160-8402 Japan; 2 Laboratory of Molecular Biomedicine for Pathogenesis, Center for Disease Biology and Integrative Medicine, Faculty of Medicine, The University of Tokyo, 7-3-1 Hongo, Bunkyo-ku, Tokyo, 113-0033 Japan

**Keywords:** Interleukins, Autoimmunity

## Abstract

Among various cytokines, interleukin (IL)-12 family cytokines have very unique characteristics in that they are composed of two distinct subunits and these subunits are shared with each other. IL-23, one of the IL-12 family cytokines, consists of p19 and p40 subunits, is mainly produced by antigen-presenting cells, and plays a critical role in the expansion and maintenance of pathogenic helper CD4^+^ T (Th)17 cells. Since we initially found that p19 is secreted in the culture supernatant of activated CD4^+^ T cells, we have further investigated the role of p19. p19 was revealed to associate with CD5 antigen-like (CD5L), which is a repressor of Th17 pathogenicity and is highly expressed in non-pathogenic Th17 cells, to form a composite p19/CD5L. This p19/CD5L was shown to activate STAT5 and enhance the differentiation into granulocyte macrophage colony-stimulating factor (GM-CSF)-producing CD4^+^ T cells. Both CD4^+^ T cell-specific conditional p19-deficient mice and complete CD5L-deficient mice showed significantly alleviated experimental autoimmune encephalomyelitis (EAE) with reduced frequency of GM-CSF^+^CD4^+^ T cells. During the course of EAE, the serum level of p19/CD5L, but not CD5L, correlated highly with the clinical symptoms. Thus, the composite p19/CD5L is a possible novel heterodimeric cytokine that contributes to EAE development with GM-CSF up-regulation.

## Introduction

Among various CD4^+^ T helper (Th) cell subsets, pro-inflammatory interleukin (IL)-17-producing Th17 cells play critical roles in the pathogenesis of inflammatory and autoimmune diseases as well as host defense and maintenance of mucosal barrier functions by secreting inflammatory cytokines such as IL-17A, IL-22, and granulocyte macrophage colony-stimulating factor (GM-CSF)^1,2^. Initially, transforming growth factor (TGF)-β1 and IL-6 were shown to induce the differentiation into Th17 cells^3–6^, but later these cells turned out to be non-pathogenic and not able to induce Th17 cell-mediated experimental autoimmune encephalomyelitis (EAE) upon adoptive transfer due to simultaneous production of anti-inflammatory cytokine IL-10^7^. Then, IL-1β, IL-6, and IL-23 were revealed to be necessary to induce the differentiation into pathogenic Th17. In particular, IL-23 is crucial for restraining IL-10 production and inducing TGF-β3 to maintain the pathogenesis of Th17 cells with high expression levels of T-bet, IL-23 receptor (R), and GM-CSF^7,8^. Interestingly, the trans-presentation of IL-6 by dendritic cell (DC)-bound IL-6Rα was recently shown to be important for generating pathogenic Th17 cells^9^. Moreover, single-cell RNA-sequence analysis of heterogeneous Th17 cells coupled with a new functional annotation approach revealed that *Gpr65*, *Toso*, and *Plzp* are genes related to promoting Th17 pathogenicity and CD5 antigen-like (CD5L) is a repressor of Th17 pathogenicity and is highly expressed in non-pathogenic Th17 cells^10,11^.

GM-CSF is a critical pro-inflammatory cytokine that is important for Th17 cell-mediated tissue inflammation^12^. GM-CSF acts on myeloid cells such as macrophages, monocytes, and DCs, but not on T cells, and these myeloid cells secrete pro-inflammatory cytokine such as IL-6 and IL-23 to sustain pathogenic Th17 cells by up-regulating IL-23R. There are various cellular sources of GM-CSF including activated T and B cells, macrophages, monocytes, endothelial cells, and others; most Th subsets produce GM-CSF^12,13^. However, it was recently demonstrated that IL-2- or IL-7-activated signal transduction and activator of transcription (STAT) 5 signaling promotes the generation of GM-CSF-producing CD4^+^ T cells, which represent a new Th cell subset, termed as ThGM^14,15^. In addition, more recent fate-mapping study identified a discrete subset of inflammation-driving Th cells regulated by IL-23 and IL-1β^16^. Specific ablation of this Th subset interrupted the inflammatory cascade and immunopathology without interfering with the entry of other Th subsets such as Th1 and Th17 into the central nerve system (CNS)^16^.

The IL-6/IL-12 family cytokines, including IL-23, are generally produced from antigen-presenting cells such as DCs and macrophages after activation^17,18^. However, in the present study, we initially noticed that p19 but not p40 is secreted into the culture supernatant from activated CD4^+^ T cells, and therefore we sought to clarify its role and mode of function. CD4^+^ T cell-specific conditional p19-deficient mice showed significantly alleviated EAE with reduced frequency of GM-CSF^+^CD4^+^ T cells in the CNS. Notably, p19 was revealed to associate with CD5L to form a composite p19/CD5L. Consistent with these results, CD5L-deficient mice also showed significantly alleviated EAE with reduced frequency of GM-CSF^+^CD4^+^ T cells. Intriguingly, during the course of EAE, the serum level of p19/CD5L but not CD5L correlated with the clinical symptoms, indicating a good diagnostic marker. Moreover, the p19/CD5L was shown to have an ability to induce cell proliferation, STAT5 phosphorylation, and augmentation of GM-CSF expression. Thus, the composite p19/CD5L would be a novel heterodimeric cytokine, which contributes to promoting the differentiation into GM-CSF-producing CD4^+^ T cells and resultant induction of EAE.

## Results

### CD4^+^ T cells secrete p19 after activation through T-cell receptor (TCR) ligation and co-stimulatory signal via CD28 enhances it

**Figure 1:**
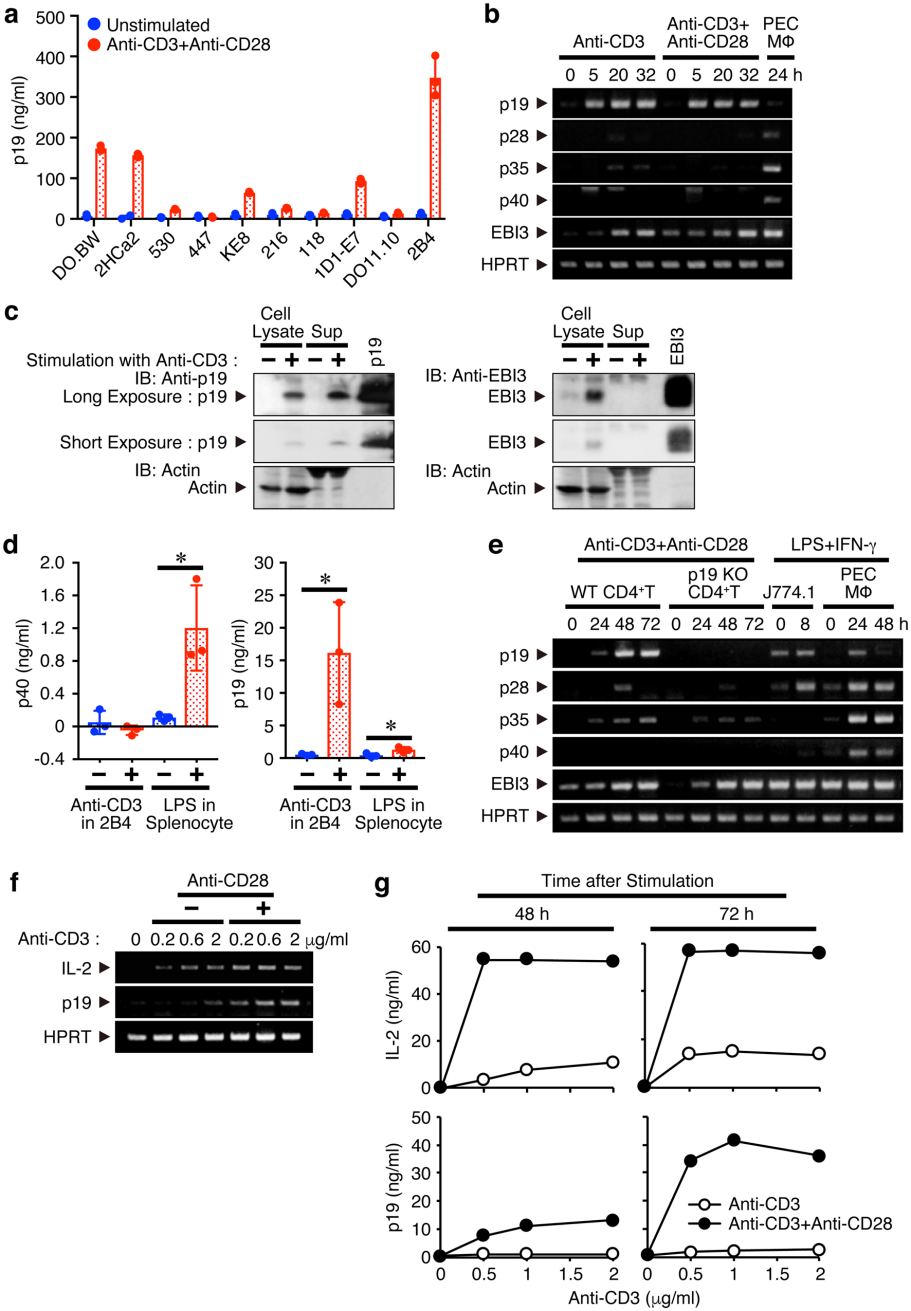
CD4^+^ T cells secret p19 after activation through TCR ligation and co-stimulatory signal via CD28 enhances it. (**a**) Mouse T cell hybridomas (DO.BW, 2HCa2, 530, 447, KE8, 216, 118, 1D1-E7, 2B4) and lymphoma (DO11.10) were stimulated with plate-bound anti-CD3 (2 μg/ml**)** and anti-CD28 (1 μg/ml**)** for 48 h, and culture supernatants were collected and analyzed for p19 by ELISA. (**b**–**d**) 2B4 cells were stimulated with plate-bound anti-CD3 in the presence or absence of anti-CD28 for indicated time points, and total RNA was extracted and analyzed for mRNA expression by semiquantitative RT-PCR (**b**). PEC macrophages prepared from mice 4 days after the injection with thioglycolate were stimulated with LPS (100 ng/ml) and IFN-γ (1000 U/ml) for 24 h and used as a positive control. Forty-eight hours later, culture supernatants were collected, cell lysates were prepared, and both were analyzed for protein expression of p19 or EBI3 by western blotting (**c**). Culture supernatants from HEK293T cells transfected with expression vectors of pCXN2-p19 or pCXN2-EBI3 were used as positive controls. Culture supernatants of the activated 2B4 cells were also analyzed for p40 together with p19 via ELISA. Culture supernatants of LPS-stimulated splenocytes were used as the positive control (**d**). (**e**) Naive CD4^+^ T cells from WT mice were stimulated with plate-bound anti-CD3 and anti-CD28 for indicated time points, and total RNA was extracted and analyzed for mRNA expression by semiquantitative RT-PCR. Mouse macrophage cell line, J774.1, and PEC macrophages stimulated with LPS and IFN-γ were used as positive controls. (**f**,**g)** Naive CD4^+^ T cells were stimulated with plate-bound anti-CD3 (0, 0.2, 0.6 and 2 μg/ml) in the presence or absence of anti-CD28 (1 μg/ml) for 48 h, and total RNA was extracted and analyzed for mRNA expression by semiquantitative RT-PCR (**f**). Culture supernatants of similarly activated naive CD4^+^ T cells with plate-bound anti-CD3 (0, 0.5, 1 and 2 μg/ml) were collected at the indicated time points and analyzed for IL-2 or p19 by ELISA (**g**). Data are representative of more than two independent experiments.

IL-23 is known to be produced from antigen-presenting cells and play a critical role in the induction and maintenance of pathogenic Th17 cells^2^. However, we initially noticed by using a p19-specific enzyme-linked immunosorbent assay (ELISA) that p19 is secreted into the culture supernatants from most of the mouse T cell hybridomas and lymphoma examined in response to stimulation with TCR ligation using anti-CD3, although the amounts secreted by the different cells varied (Fig. 1a). The 2B4 T cell hybridoma secreted p19 most efficiently, so we next examined the up-regulation of p19 at the mRNA level in the 2B4 cells by semiquantitative RT-PCR. After stimulation with plate-coated anti-CD3 in the presence or absence of anti-CD28, p19 mRNA expression was greatly enhanced irrespective of CD28 signaling (Fig. 1b). Similarly, EBI3 mRNA expression was also enhanced, but the mRNA expression of other IL-6/IL-12 family-related subunits such as p28, p35, and p40 was hardly augmented. Western blot analysis also revealed that stimulation of 2B4 cells with plate-coated anti-CD3 increased the p19 expression not only in the cell lysates but also in the culture supernatants (Fig. 1c). However, EBI3 expression was detected in the cell lysates but not in the culture supernatants, implying that the possible association between p19 and EBI3 in the culture supernatants of activated T cells would be negligible. ELISA results confirmed that no p40 was secreted in the culture supernatant (Fig. 1d). Next, we used primary naive CD4^+^ T cells purified from spleen cells instead of T cell lines. Consistent with the results obtained using 2B4 cells, mRNA expression of both p19 and EBI3 was greatly enhanced, but the mRNA expression of other subunits including p28, p35, and p40 was scarcely augmented (Fig. 1e). Then, the necessity of a co-stimulatory signal through CD28, which is well known to be indispensable for IL-2 production, was examined. Co-stimulation through CD28 greatly augmented the mRNA expression of p19, but less efficiently than that of IL-2 (Fig. 1f). Similar results were obtained when the culture supernatants were analyzed for p19 and IL-2 by ELISA (Fig. 1g).

Collectively, these results suggest that CD4^+^ T cells secrete p19 after activation through TCR ligation and that co-stimulatory signal via CD28 is necessary for its optimal production.

### CD4^+^ T cell-specific conditional p19-deficient mice show alleviated EAE with reduced frequency of GM-CSF^+^CD4^+^ T cells in the CNS

**Figure 2:**
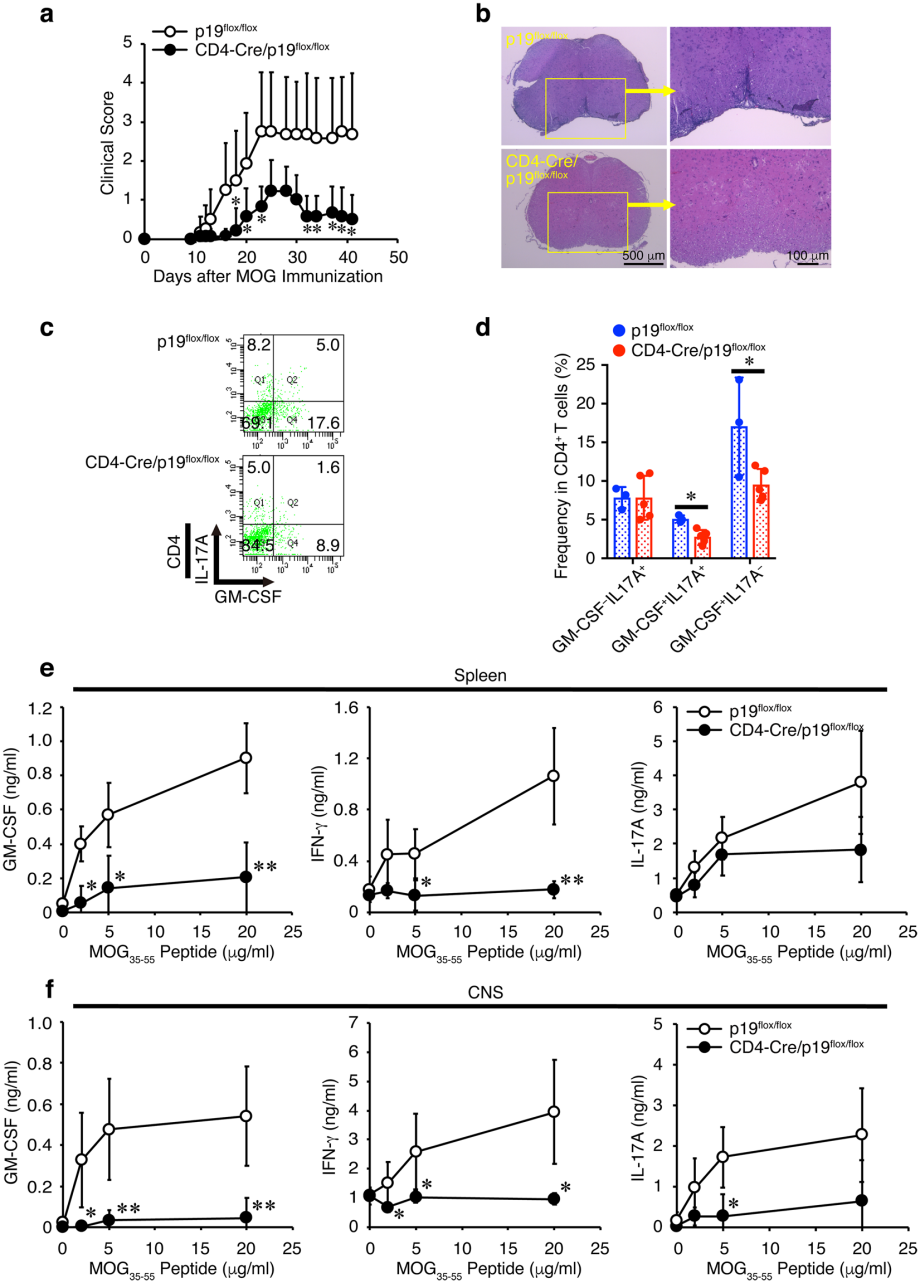
CD4^+^ T cell-specific conditional p19-deficient mice show alleviated EAE with reduced frequency of GM-CSF^+^CD4^+^ T cells in the CNS. Control p19^flox/flox^ mice or CD4^+^ T-cell-specific conditional 19-deficient (CD4-Cre/p19^flox/flox^) mice were immunized with MOG_35-55_ peptide and their clinical scores were monitored with time (**a**). On day 14, spinal cords and brains were harvested, and the CNS was histopathologically analyzed with H&E staining. Representative images are shown (**b**). On day 41, mononuclear cells were isolated from the CNS, and intracellular cytokine staining was performed after restimulation with PMA and ionomycin. Representative dot plots of GM-CSF and IL-17A in CD4^+^ T cells are shown (**c**). Average frequencies of respective CD4^+^ T cells were calculated and compared (**d**). Response to the recall antigen MOG_35-55_ peptide was examined using spleen cells (**e**) and mononuclear cells infiltrating the CNS (**f**) on day 14. The culture supernatants were analyzed via ELISA for cytokine production as indicated. Data are shown as mean ± SD (n = 3–7) and are representative of two independent experiments. *P* values were determined using unpaired, two-tailed Student’s *t*-test. *P < 0.05, **P < 0.01.

Next, the role of p19 secreted from activated CD4^+^ T cells was investigated. Because completely p19-deficient mice are resistant to the development of EAE^19^, we generated CD4^+^ T cell-specific conditional p19-deficient mice by mating CD4-Cre-Tg mice and p19^flox/flox^ mice^20^. Using the conditional p19-deficient mice allowed us to explore the role of p19 in CD4^+^ T cells in the susceptibility to EAE. The CD4^+^ T cell-specific conditional p19-deficient mice showed greatly alleviated clinical scores compared with control p19^flox/flox^ mice (Fig. 2a, Supplementary Table 1). Mononuclear cell infiltration into the spinal cord was observed to be much less intense in the conditional p19-deficient mice compared with that in control p19^flox/flox^ mice (Fig. 2b). To examine the molecular mechanism whereby the p19 promotes the development of EAE, fluorescence-activated cell sorting (FACS) analysis was performed to see the frequency of cytokine-producing CD4^+^ T cells in mononuclear cells infiltrating in the CNS. The frequency of GM-CSF^+^CD4^+^ T cells but not IL-17A^+^GM-CSF^−^CD4^+^ T cells in the CNS was significantly reduced in CD4^+^ T-cell-specific conditional p19-deficient mice compared with that in control p19^flox/flox^ mice (Fig. 2c, d). To examine the response to the recall antigen MOG_35-55_ peptide, splenic and mononuclear cells infiltrating the CNS were restimulated with MOG_35-55_ peptide in vitro, and their culture supernatants were analyzed for GM-CSF, IFN-γ and IL-17A production (Fig. 2e, f). GM-CSF and IFN-γ production were greatly reduced in the splenic and mononuclear cells infiltrating the CNS of CD4^+^ T-cell-specific conditional p19-deficient mice. Additionally, IL-17A production was decreased in the mononuclear cells but not in the splenic cells.

These results suggest that p19 expressed in CD4^+^ T cells also plays a critical role in the promotion of development of EAE, possibly through up-regulating GM-CSF.

### Differentiation into GM-CSF-producing CD4^+^ T cells is impaired in p19-deficient CD4^+^ T cells in vitro

**Figure 3:**
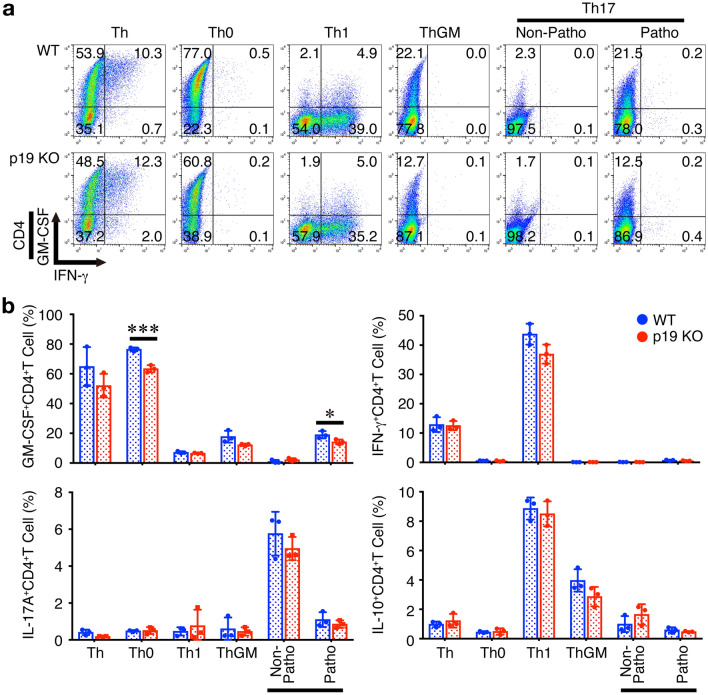
Differentiation into GM-CSF-producing CD4^+^ T cells is impaired in p19-deficient CD4^+^ T cells in vitro. Naive CD4^+^ T cells from WT mice or complete p19-deficient mice (p19 KO) were stimulated with plate-coated anti-CD3 (2 μg/ml) and anti-CD28 (1 μg/ml) for 4 days under various Th-polarizing conditions; Th, Th0, Th1, ThGM, nonpathogenic Th17, and pathogenic Th17. These cells were then restimulated with PMA and ionomycin, and the intracellular cytokine staining was performed. Representative dot plots for GM-CSF, IL-17A, IFN-γ, and IL-10 in CD4^+^ T cells are shown (**a**), and average frequencies of respective CD4^+^ T cells were calculated and compared (**b**). Data are shown as mean ± SD (n = 3) and are representative of three independent experiments. *P* values were determined using unpaired, two-tailed Student’s *t*-test. *P < 0.05, ***P < 0.001.

Several Th subsets including Th1, Th2, and Th17 are known to have a potential to produce GM-CSF^13^ and a subset of GM-CSF-producing Th cells termed ThGM was also identified and reported to be regulated by IL-7/STAT5 signaling^14^. A recent fate-mapping study on GM-CSF expression identified a subset of inflammation-driving Th cells in EAE, which requires IL-23R and IL-1R but is distinct from Th1 and Th17 subsets^16^. Therefore, we next examined the effects of p19-deficiency in naive CD4^+^ T cells on differentiation into various Th subsets in vitro using complete p19-deficient mice (p19 KO). As previously reported, GM-CSF^+^CD4^+^ T cells were generated under various Th-polarizing conditions, although the frequency was varied among them^13^. In our conditions, a higher frequency of GM-CSF^+^CD4^+^ T cells was observed in Th and Th0 conditions (Fig. 3a, b). The frequency of GM-CSF^+^CD4^+^ T cells under Th0 and pathogenic Th17-polarizing conditions was significantly decreased in p19-deficient CD4^+^ T cells compared with that in wild-type (WT) CD4^+^ T cells (Fig. 3a, b). A similar tendency of the frequency of GM-CSF^+^CD4^+^ T cells to decrease under Th and ThGM-polarizing conditions were also observed, although they were not constantly significant. No other significant difference in the frequencies of IFN-γ^+^CD4^+^ T cells, IL-17A^+^CD4^+^ T cells, and IL-10^+^CD4^+^ T cells were observed between WT and p19-deficient CD4^+^ T cells under any Th-polarizing conditions.

Thus, p19 in CD4^+^ T cells plays a pivotal role in promoting differentiation into GM-CSF^+^CD4^+^ T cells under Th0 and pathogenic Th17-polarizing conditions and tends to promote this differentiation under Th and ThGM-polarizing conditions.

### p19 associates with CD5L to form a possible heterodimer of p19/CD5L

**Figure 4:**
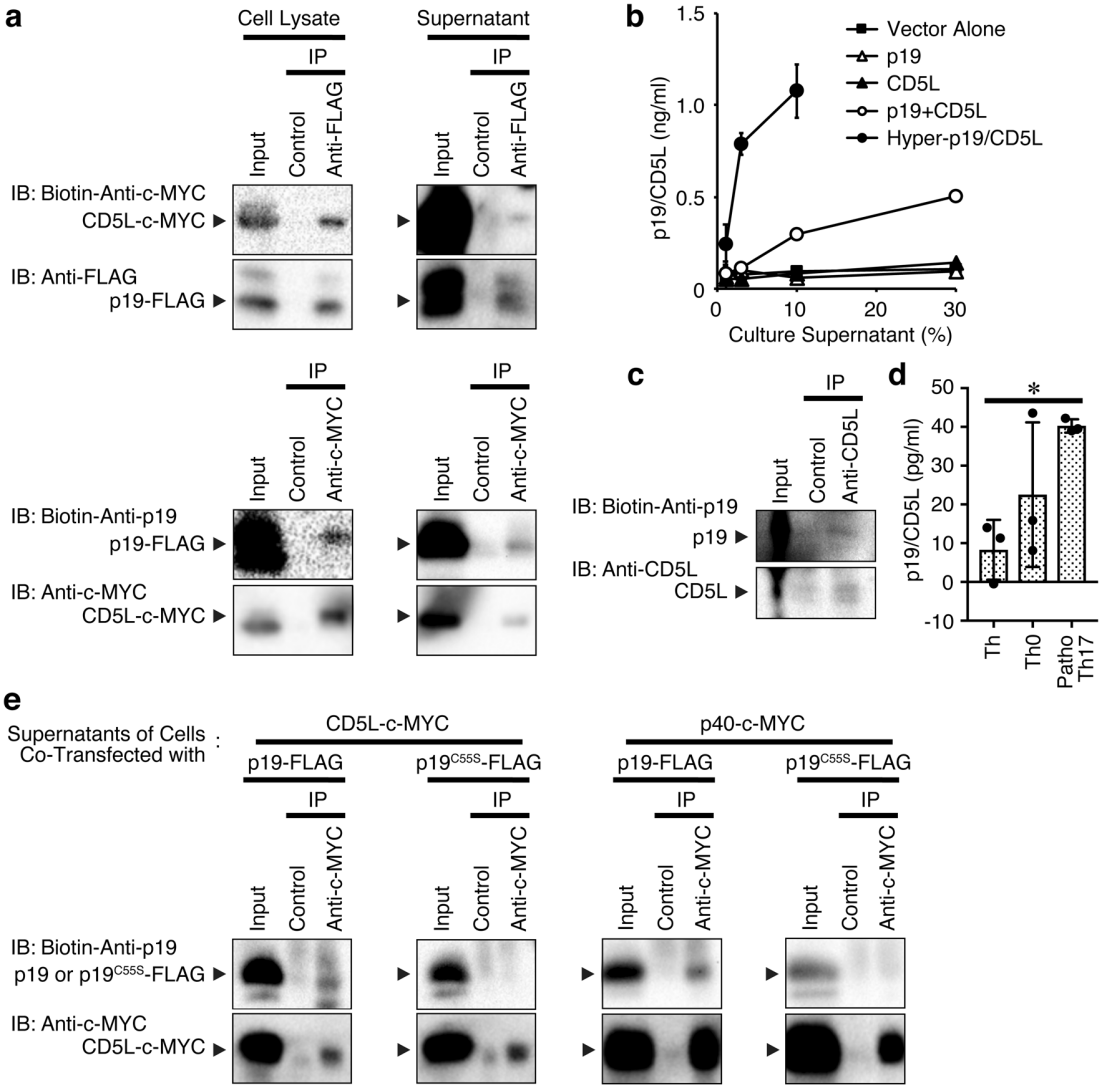
p19 associates with CD5L to form a putative heterodimer of p19/CD5L. (**a**) HEK293-F cells were transiently cotransfected with p3 × FLAG-CMV-14-p19 and pCMV3-CD5L-c-MYC and cultured for 3 days, and total cell lysates or culture supernatants were immunoprecipitated with anti-FLAG or anti-c-MYC and control antibody followed by western blotting with biotin-conjugated anti-c-MYC or biotin-conjugated anti-p19, respectively. Immunoprecipitated protein was confirmed by western blotting with the antibody used for immunoprecipitation. (**b**) HEK293T cells were transiently transfected with the control vector p3 × FLAG-CMV-14 alone, p3 × FLAG-CMV-14-p19, pCMV3-CD5L-c-MYC, or both and p3 × FLAG-CMV-14-Hyper-p19/CD5L, then cultured for 3 days. The culture supernatants were then subjected to p19/CD5L-specific ELISA in triplicate. Data are shown as the mean ± SD. (**c**) Naive CD4^+^ T cells were stimulated with plate-coated anti-CD3 (5 μg/ml) and anti-CD28 (2 μg/ml) under pathogenic Th17-polarizing conditions for 3 days. Resultant culture supernatants were collected and concentrated by centrifugation using a centrifugal filter approximately 8 times, and subjected to immunoprecipitation with anti-CD5L followed by western blotting with biotin-conjugated anti-p19 and subsequently anti-CD5L. (**d**) Naive CD4^+^ T cells were also similarly stimulated with plate-coated anti-CD3 and anti-CD28 under Th, Th0 and pathogenic Th17-polarizing conditions for 3 days. Resultant culture supernatants were collected and concentrated by centrifugation using a centrifugal filter approximately 10 times, followed by p19/CD5L-specific ELISA in triplicate. (**e**) HEK293-F cells were transiently cotransfected with p3 × FLAG-CMV-14-p19^WT^ or p3 × FLAG-CMV-14-p19^C55S^ and pCMV3-CD5L-c-MYC and cultured for 3 days. The culture supernatants were immunoprecipitated with anti-c-MYC and control antibody followed by western blotting with biotin-conjugated anti-p19, then anti-c-MYC. Data are shown as the mean ± SD and representative of four (**a**) or two (**b**–**e**) independent experiments. *P* values were determined using one-way ANOVA. *P < 0.05.

In investigating the molecular mechanism by which p19 augments the differentiation into GM-CSF^+^CD4^+^ T cells, western blot analysis revealed that p19 forms a homodimer in the culture supernatants of human embryonic kidney (HEK) 293 T cells transfected with a p19 expression vector under non-reducing conditions (Supplementary Fig. 1). However, this culture supernatant failed to act as a biological cytokine (see later). While attempting to identify any biological activity of the p19 independently of the common p40 subunit of IL-12 and IL-23, we found an interesting study reporting the formation of a heterodimer between p40 and CD5L, although the bioactivity of the heterodimer p40/CD5L remains unknown^21^. This paper prompted us to conceive of a possible association between the CD5L and p19. First of all, immunoprecipitation analysis followed by western blotting of culture supernatants of HEK293-F cells transiently transfected with the expression vectors for p19-FLAG and CD5L-c-MYC indeed revealed the association between them not only in the cell lysates but also in the culture supernatants (Fig. 4a). We then established a p19/CD5L-specific sandwich ELISA system using commercially available CD5L-specific and biotin-conjugated p19-specific antibodies. Culture supernatants of HEK293T cells transiently transfected with expression vectors for either p19-FLAG or CD5L-c-MYC alone, both p19-FLAG and CD5L-c-MYC together, and a single-chain p19/pCD5L designated hyper-p19/CD5L were analyzed using the sandwich ELISA. The ELISA efficiently detected the culture supernatants of the hyper-p19/pCD5L-transfected cells but not those of either the p19- or CD5L-transfected cells (Fig. 4b). Notably, the culture supernatant of the cells cotransfected with the p19 and CD5L expression vectors was less than that for hyper-p19/pCD5L but was significantly detected by the sandwich ELISA. Similarly, immunoprecipitation followed by western blotting revealed a significant association between p19 and CD5L in the concentrated culture supernatant of activated WT CD4^+^ T cells under pathogenic Th17-polarizing conditions (Fig. 4c). ELISA also showed an association between p19 and CD5L under Th, Th0 and pathogenic Th17-polarizing conditions (Fig. 4d).

Heterodimeric interaction between the p19 and p40 subunits in the human IL-23 complex is reportedly stabilized by an intermolecular disulfide bond between the p19 Cys54 and p40 Cys177 residues^22,23^. Because the mature human p19 Cys54 corresponds to mature mouse p19 Cys55^24^, we constructed the mouse p19 mutant, C55S (TGT → AGT), in which Ser replaced the disulfide bond-forming cysteine, Cyc55. The ability to bind to CD5L as well as to p40 as a control was compared between WT p19 (p19^WT^) and the p19 mutant C55S (p19^C55S^) via immunoprecipitation followed by western blotting of culture supernatants of HEK293-F cells transiently transfected with the WT p19-FLAG or p19 mutant C55S-FLAG and CD5L-c-MYC or p40-c-MYC expression vectors (Fig. 4e). As expected, the p19 mutant C55S, but not the WT p19, failed to bind to p40. Likewise, the p19 mutant also lost the ability to bind to CD5L, although WT p19 bound to CD5L, indicating that Cys55 in p19 is important for forming heterodimers with CD5L as well as p40.

These results suggest that p19 forms a cytokine-like composite with CD5L in the culture supernatants of activated CD4^+^ T cells as well as those of HEK293T cells over-expressing both p19 and CD5L.

### CD5L-deficient mice show alleviated EAE with reduced frequency of GM-CSF^+^CD4^+^ T cells in the CNS

**Figure 5:**
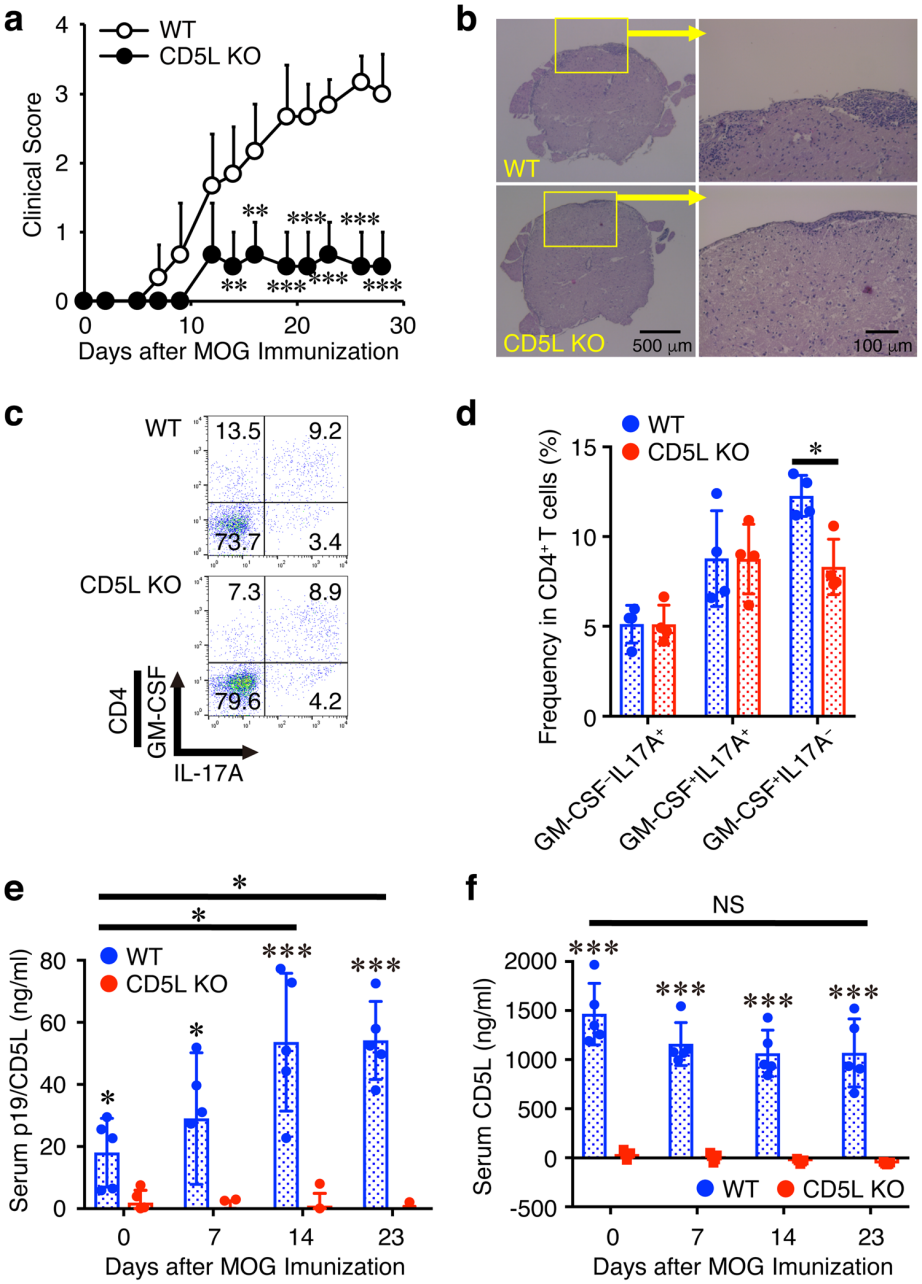
CD5L-deficient mice show alleviated EAE with reduced frequency of GM-CSF^+^CD4^+^ T cells in the CNS. (**a**–**d**) WT mice or CD5L-deficient mice were immunized with MOG_35-55_ peptide and their clinical scores were monitored with time (**a**). On day 15, spinal cords and brains were harvested, and the CNS was histopathologically analyzed with H&E staining. Representative images are shown (**b**). Mononuclear cells were also isolated from the CNS, and intracellular cytokine staining was performed after restimulation with PMA and ionomycin. Representative dot plots of GM-CSF, and IL-17A in CD4^+^ T cells are shown (**c**). Average frequencies of respective CD4^+^ T cells were calculated and compared (**d**). Blood was also taken over time and serum levels of p19/CD5L and CD5L were determined by ELISA (**e**, **f**). Data are shown as mean ± SD (n = 4–6) and are representative of three independent experiments. *P* values were determined using unpaired, two-tailed Student’s *t*-test (**a**, **d**) or one-way ANOVA (**e**, **f**). *P < 0.05, **P < 0.01, ***P < 0.001. NS, not significant.

Because CD5L was previously reported to restrain Th17 cell pathogenicity by regulating lipid biosynthesis^11^ and no CD4^+^ T cell-specific conditional CD5L-deficient mice were available, next the susceptibility of complete CD5L-deficient mice to EAE was explored. Similar to the results with CD4^+^ T cell-specific conditional p19-deficient mice (Fig. 2a), CD5L-deficient mice showed greatly delayed onset of EAE and alleviated clinical scores compared with WT mice (Fig. 5a, Supplementary Table 2). Much less intense mononuclear cell infiltration into the spinal cord was also observed in the CD5L-deficient mice compared with that in WT mice (Fig. 5b). Moreover, FACS analysis revealed that the frequency of GM-CSF^+^IL-17A^−^CD4^+^ T cells but not GM-CSF^−^IL-17A^+^CD4^+^ T cells nor GM-CSF^+^IL-17A^+^CD4^+^ T cells in the CNS was significantly reduced in CD5L-deficient mice compared with that in WT mice (Fig. 5c, d). During EAE development, the serum p19/CD5L level was determined by specific ELISA and revealed to be greatly enhanced as the EAE clinical signs progressed in WT mice (Fig. 5e). Conversely, the CD5L serum level, which was > 1 μg/ml and was much higher than the p19/CD5L level in the WT mice, was minimally varied (Fig. 5f). Serum levels of both p19/CD5L and CD5L were negligible in CD5L-deficient mice.

These results suggest that CD5L-deficient mice show alleviated EAE with reduced frequency of GM-CSF^+^IL-17A^−^CD4^+^ T cells in the CNS, and that a serum level of p19/CD5L in WT mice well corelates with the progression of EAE.

### Differentiation into GM-CSF-producing CD4^+^ T cells is impaired in CD5L-deficient CD4^+^ T cells in vitro

**Figure 6:**
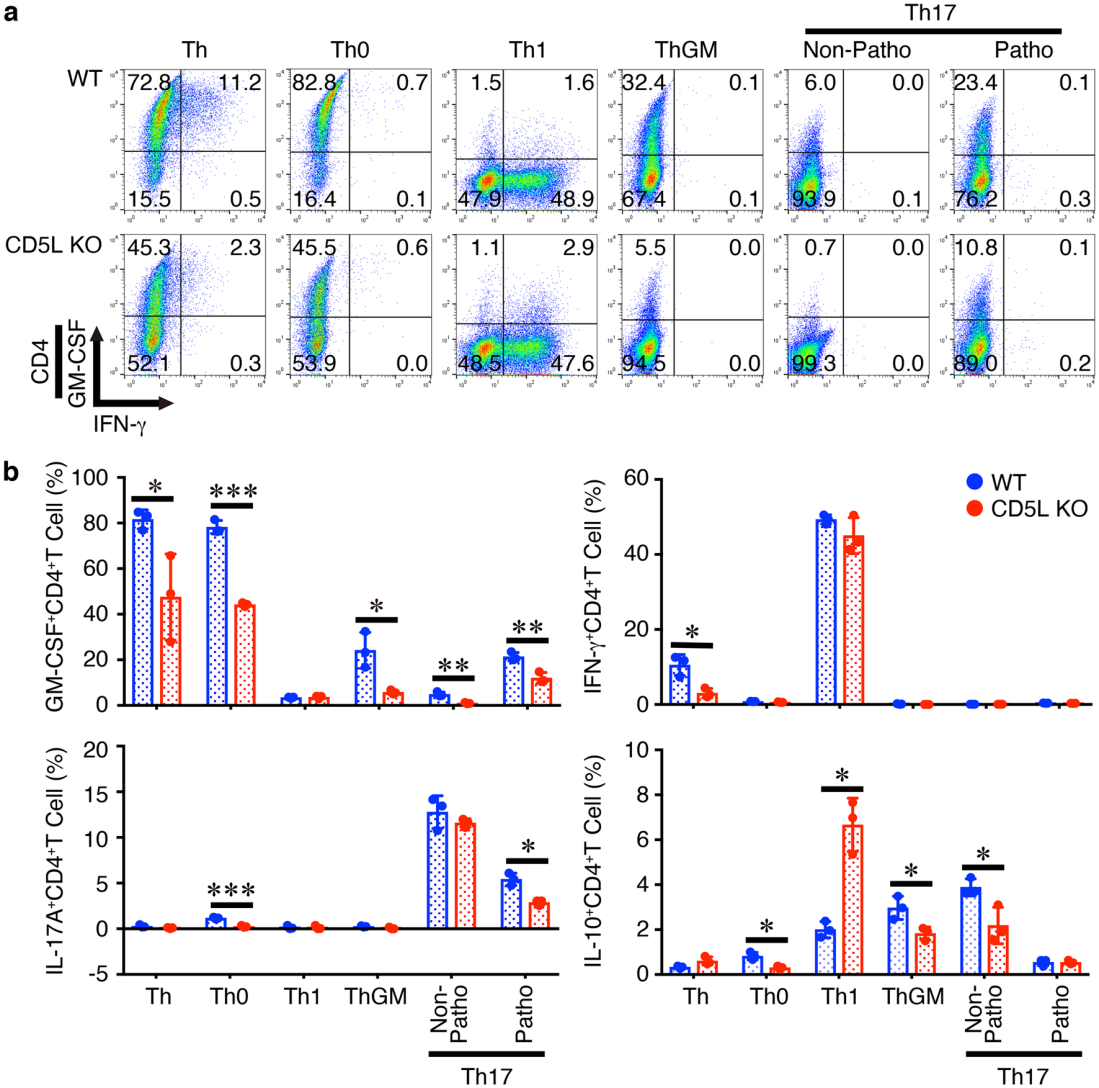
Differentiation into GM-CSF-producing CD4^+^ T cells is impaired in CD5L-deficient CD4^+^ T cells in vitro. Naive CD4^+^ T cells from WT mice or CD5L-deficient mice were stimulated with plate-coated anti-CD3 (2 μg/ml) and anti-CD28 (1 μg/ml) for 4 days under various Th-polarizing conditions; Th, Th0, Th1, ThGM, non-pathogenic Th17, and pathogenic Th17. These cells were then restimulated with PMA and ionomycin, and the intracellular cytokine staining was performed. Representative dot plots for GM-CSF, IL-17A, IFN-γ, and IL-10 in CD4^+^ T cells are shown (**a**), and average frequencies of respective CD4^+^ T cells were calculated and compared (**b**). Data are shown as mean ± SD (n = 3) and are representative of three independent experiments. *P* values were determined using unpaired, two-tailed Student’s *t*-test. *P < 0.05, **P < 0.01, ***P < 0.001.

To further explore the role of CD5L in CD4^+^ T cells, we next examined the effects of CD5L deficiency in naive CD4^+^ T cells on differentiation into various Th subsets in vitro using CD5L-deficient CD4^+^ T cells. Similar to the results obtained using the CD4^+^ T cell-specific conditional p19-deficient mice (Fig. 2), the frequency of GM-CSF^+^CD4^+^ T cells under Th-polarizing conditions such as Th, Th0, ThGM, and non-pathogenic and pathogenic Th17 were significantly reduced in CD5L-deficient CD4^+^ T cells compared with that in WT CD4^+^ T cells (Fig. 6a, b). Similar reduction in the frequencies of IL-17A^+^CD4^+^ T cells under Th0 and pathogenic Th17-polarizing conditions was observed in CD5L-deficient CD4^+^ T cells compared with that in WT CD4^+^ T cells. In addition, the frequencies of some other CD4^+^ T cells including IFN-γ^+^CD4^+^ T cells under Th condition and IL-10^+^CD4^+^ T cells under Th0, ThGM-, and non-pathogenic Th17-polarizing conditions were also decreased. Conversely, the frequencies of IL-10^+^CD4^+^ T cells under Th1-polarizing conditions tend to consistently increase in CD5L-deficient CD4^+^ T cells.

These results suggest that, similar to the results using p19-deficient CD4^+^ T cells (Fig. 2), CD5L in CD4^+^ T cells plays a pivotal role in promoting the differentiation into GM-CSF^+^CD4^+^ T cells under Th0 and pathogenic Th17-polarizing conditions, and also under Th and ThGM-polarizing conditions.

### Correlation of p19, CD5L, p19/CD5L, and GM-CSF under various Th-polarizing conditions

We next explored the expression levels of p19, CD5L, p19/CD5L, and GM-CSF using real-time RT-PCR (Supplementary Fig. 2a), ELISA (Fig. 4d, Supplementary Fig. 2b), and intracellular staining with FACS (Supplementary Fig. 2c) after activation of naive CD4^+^ T cells via plate-coated anti-CD3 and anti-CD28 under various Th-polarizing conditions. Overall, the p19 expression at mRNA and protein levels was detected in almost all of CD4^+^ T cells activated under Th-polarizing conditions except for non-pathogenic Th17-polarizing conditions (Supplementary Fig. 2a–d). The lowest expression of p19 under non-pathogenic Th17-polarizing conditions would be presumably due to the suppressive effect of TGF-β1 on p19 expression, although further studies are necessary to clarify this. The CD5L expression under non-pathogenic Th17-polarizing conditions have a tendency to be higher than other conditions including pathogenic Th17-polarizing conditions (Supplementary Fig. 2a–d), which is consistent with a previous report^11^. GM-CSF expression was higher under Th-, Th0-, ThGM-, and pathogenic Th17-polarizing conditions (Supplementary Fig. 2a,b). This pattern seems to be roughly analogous to that of p19/CD5L expression, which was detected as CD4^+^ T cells double-positive for p19 and CD5L by intracellular staining, although the expression level was very low (Supplementary Fig. 2c,d). p19/CD5L-specific ELISA revealed a similar tendency in the concentrated culture supernatants of CD4^+^ T cells activated under Th-, Th0- and pathogenic Th17-polarizing conditions (Fig. 4d).

These results further support the correlated expression between p19/CD5L and GM-CSF.

### p19/CD5L but not either alone efficiently induces cell proliferation, STAT5 phosphorylation, and augmentation of GM-CSF expression

**Figure 7:**
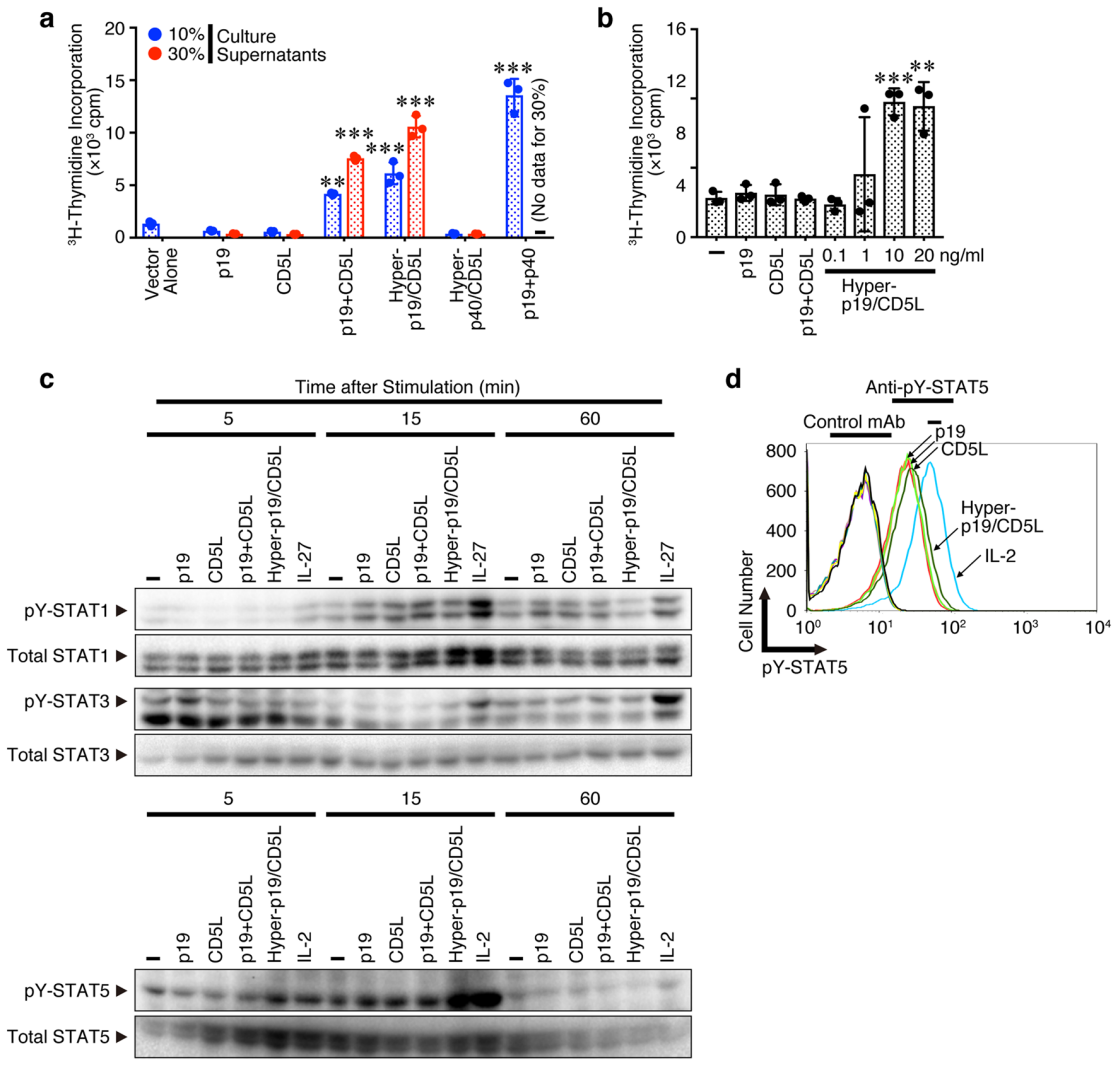
p19/CD5L but not either alone efficiently induces cell proliferation, STAT5 phosphorylation. (**a**,**b**) HEK293T cells were transfected with the control vector p3 × FLAG-CMV-14 alone, p3 × FLAG-CMV-14-p19, pCMV3-CD5L-c-MYC, p3 × FLAG-CMV-14-hyper-p19/CD5L, and p3 × FLAG-CMV-13-hyper-p40/CD5L. The cells were also cotransfected with expression vectors of p3 × FLAG-CMV-14-p19 and pCMV3-CD5L-c-MYC, together with those of p3 × FLAG-CMV-14-p19 and p3 × FLAG-CMV-14-p40, which produced IL-23 as the positive control. The total amount of DNA in each transfection sample was adjusted to be kept equal with the empty vector. Three days later, culture supernatants were collected, and 10% or 30% of them were used for stimulation of Ba/F3 cells expressing gp130/IL-12Rβ1/IL-12Rβ2/IL-23Rα. For p19 + p40 (IL-23), only 10% culture supernatant was added; therefore, “**–**” means that no data exist for the 30% lane (**a**). Ba/F3 cells were also stimulated with purified recombinant p19, CD5L, a mixture of p19 and CD5L (all 20 ng/ml), and hyper-p19/CD5L (0.1**–**20 ng/ml) (**b**). Proliferative activity of these cells was determined by measuring ^3^H-thymidine incorporated into the DNA. (**c**,**d**) Naive CD4^+^ T cells from WT mice were stimulated with plate-coated anti-CD3 (2 μg/ml**)** and anti-CD28 (1 μg/ml**)** for 3 days under Th conditions, washed, and rested in 10% FBS medium for 6 h. These cells were unrestimulated (**–**) or then restimulated with purified recombinant p19, CD5L, a mixture of p19 and CD5L, hyper-p19/CD5L, IL-27 (positive control for phosphorylation of STAT1 and STAT3, all 20 ng/ml), or IL-2 (positive control for phosphorylation of STAT5, 100 U/ml) for 5, 15 and 60 min, and subjected to western blotting with anti-pY-STATs and subsequently anti-total STATs (**c**). FACS analysis was also performed using anti-pY-STAT5 and its control antibody after stimulation for 20 min (**d**). Data are shown as the mean ± SD in triplicate and are representative of three (**a**, **c**, **d**) or two (**b**) independent experiments. *P* values were determined using one-way ANOVA. *P < 0.05, **P < 0.01, ***P < 0.001.

If p19/CD5L could be a bioactive cytokine, it should induce proliferation of a factor-dependent mouse pro-B cell line Ba/F3 expressing appropriate receptors for it and also phosphorylation of appropriate STAT. Because we lack knowledge of the p19/CD5L receptor, we used Ba/F3 cells expressing nearly all receptor subunits available for the IL-12 family cytokines, gp130, IL-12Rβ1, IL-12Rβ2, IL-23Rα, and endogenous WSX-1, which are responsible to IL-12, IL-23, and IL-27 (Supplementary Fig. 3). Culture supernatants of HEK293T cells transfected with the expression vectors for p19, CD5L, p40, hyper-p19/CD5L, and hyper-p40/CD5L^21^ and those cotransfected with the expression vectors for p19 and CD5L as well as for p19 and p40 were used to stimulate the Ba/F3 cells. Importantly, the culture supernatant of cells cotransfected with expression vectors for p19 and CD5L together efficiently induced the similar proliferation to that for p19 and p40-forming IL-23 (Fig. 7a). However, that for p19 and CD5L alone failed to induce this proliferation, although p19 appeared to form a homodimer (Supplementary Fig. 1). Additionally, culture supernatants of cells transfected with the expression vector for hyper-p19/CD5L but not for hyper-p40/CD5L also induced proliferation. Similar experiments were performed using recombinant p19, CD5L, hyper-p19/CD5L and IL-23. Consistently, purified recombinant hyper-p19/CD5L dose-dependently induced Ba/F3 cell proliferation (Fig. 7b). A mixture of only recombinant p19 and CD5L did not induce proliferation, suggesting that the synergistic effect between individual p19 and CD5L does not cause hyper-p19/CD5L-induced proliferation. Next, we used CD4^+^ T cells activated under Th conditions to examine whether purified recombinant hyper-p19/CD5L induces STAT phosphorylation because these activated CD4^+^ T cells appeared to express IL-12, IL-23 and IL-27 receptors (Supplementary Fig. 4). Interestingly, western blot analysis revealed that hyper-p19/CD5L greatly induced phosphorylation of STAT5 in a time dependent-manner but not STAT1 and STAT3, while neither p19 nor CD5L alone induced STAT5 phosphorylation (Fig. 7c). Again, the mixture of only recombinant p19 and CD5L failed to induce phosphorylation. FACS analysis also revealed that p19/CD5L together, but not alone, similarly augmented STAT5 phosphorylation (Fig. 7d). These results are consistent with the importance of STAT5 for differentiation into ThGM^14^.

**Figure 8:**
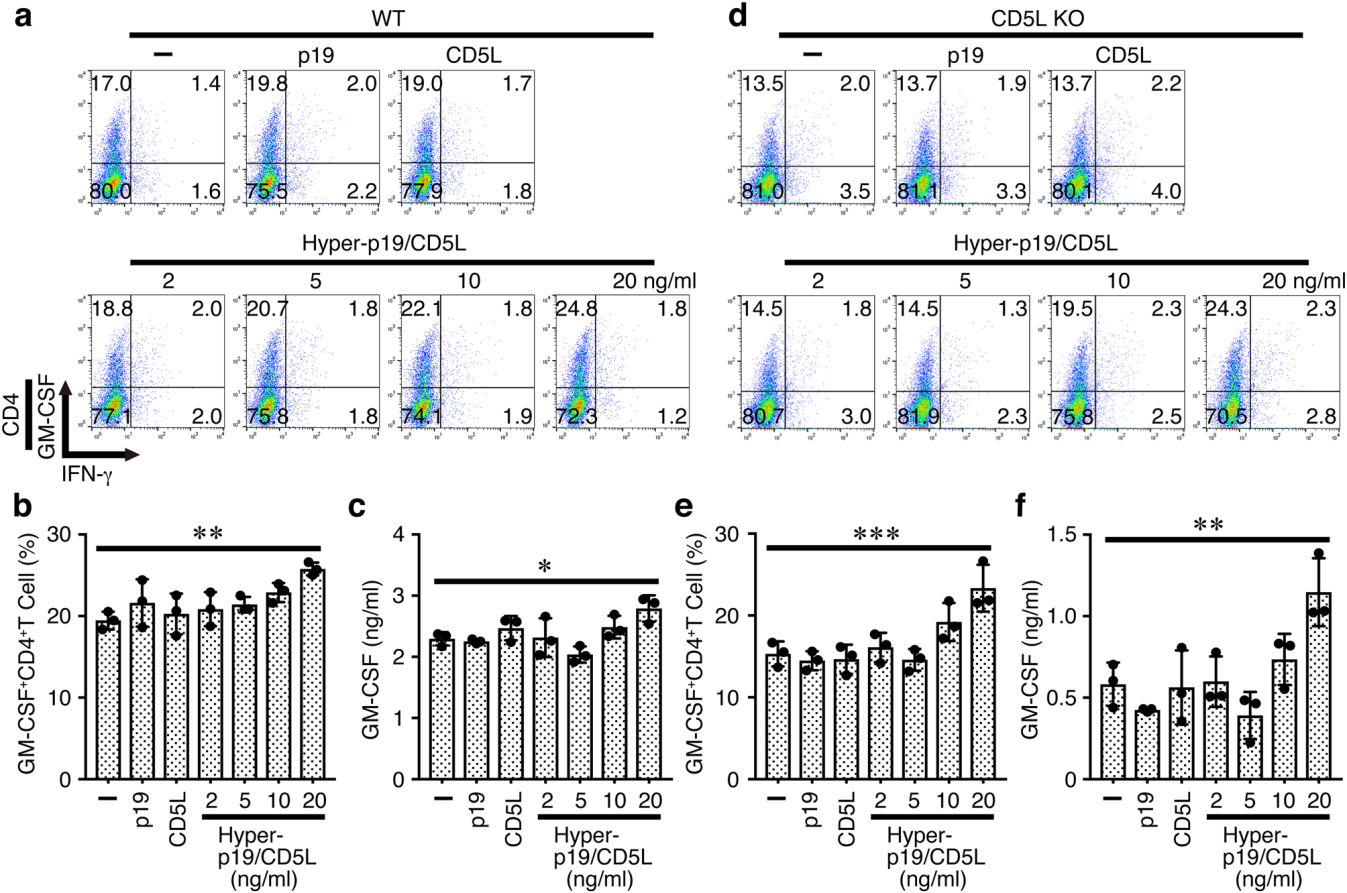
p19/CD5L but not either alone induces augmentation of GM-CSF expression. Naive CD4^+^ T cells from WT mice were stimulated with plate-coated anti-CD3 (2 μg/ml) and anti-CD28 (1 μg/ml) in the presence of recombinant p19, CD5L (all 20 ng/ml), and hyper-p19/CD5L (2–20 ng/ml) under Th conditions for 3 days, and subjected to intracellular staining of GM-CSF and IFN-γ (**a**, **b**). Representative dot plots were shown (**a**). Culture supernatants were analyzed for GM-CSF by ELISA (**c**). Similarly, naive CD4^+^ T cells from CD5L-deficient mice were stimulated and subjected to intracellular staining of GM-CSF and IFN-γ (**d**, **e**). Representative dot plots were shown (**d**). Culture supernatants were analyzed for GM-CSF by ELISA (**f**). Data are shown as mean ± SD in triplicates and are representative of more than two independent experiments. *P* values were determined using one-way ANOVA. *P < 0.05, **P < 0.01, ***P < 0.001.

Finally, we explored whether recombinant p19/CD5L protein can augment the GM-CSF expression by intracellular staining and ELISA after stimulation of naive CD4^+^ T cells with plate-coated anti-CD3 and anti-CD28 under Th conditions. The p19/CD5L slightly (but dose-dependently) augmented the frequency of GM-CSF^+^CD4^+^ T cells (Fig. 8a, b). In contrast, p19 and CD5L alone failed to augment it. Similar augmentation of GM-CSF production was observed in the culture supernatants, which were detected by ELISA (Fig. 8c). Because the effect of p19/CD5L on GM-CSF up-regulation in WT CD4^+^ T cells is not profound, we next used CD5L-deficient naive CD4^+^ T cells. Although these CD4^+^ T cells still increased the expression of GM-CSF and its production into the culture supernatants even without addition of p19/CD5L, the p19/CD5L further augmented the expression and production of GM-CSF (Fig. 8d–f).

Thus, p19/CD5L, but neither p19 nor CD5L alone, efficiently induces cell proliferation, STAT5 phosphorylation, and augmentation of GM-CSF expression, indicating that p19/CD5L is possibly a bioactive cytokine-like molecule.

## Discussion

IL-6/IL-12 family cytokines including IL-23, IL-27, IL-35, and IL-39 play pivotal roles in the differentiation of CD4^+^ T cells into effector Th cells and the exertion of their effector functions^17,18,25,26^. Among various cytokines, this family of cytokines has very unique characteristics in that they are composed of two distinct subunits and these subunits are shared with each other^26^. Therefore, there is still a great potential to identify new heterodimeric cytokines by just changing the combination of two subunits^26,27^. The IL-6/IL-12 family cytokines are generally produced from antigen-presenting cells such as DCs and macrophages after activation. However, we initially found that p19 but not p40 is secreted into the culture supernatant from activated CD4^+^ T cells (Fig. 1), which is consistent with the results of real-time RT-PCR analysis in the first cloning paper of p19 showing enhanced mRNA expression of p19 in Th1 and Th2 cells^24^. Notably, in the present study, we found that p19 associated with CD5L to form a possible new composite heterodimer of p19/CD5L (Fig. 4) that could induce cell proliferation (Fig. 7a,b), STAT5 phosphorylation (Fig. 7c,d), and augmentation of GM-CSF expression (Figs. 2, 3, 5, 6, 8, Supplementary Fig. 2). Moreover, a p19/CD5L-specific sandwich ELISA established using antibodies to respective subunits (Fig. 4b) could detect the p19/CD5L in the serum during the development of EAE (Fig. 5e). Interestingly, a similar heterodimer of CD5L and p40 forming a p40/CD5L complex, has also been reported, but its biological activity remains unknown^21^. The results showing that CD4^+^ T cell-specific conditional p19-deficient mice showed attenuated EAE development (Fig. 2) and that p19 alone was not functional (Fig. 7, 8) highly suggest that CD4^+^ T cells are the source of p19/CD5L in vivo and that p19/CD5L is responsible for the p19 phenotype. Consistently, analyses using immunoprecipitation followed by western blotting and p19/CD5L-specific ELISA revealed the presence of a possible p19 and CD5L heterodimer in the culture supernatant of CD4^+^ T cells activated in vitro (Fig. 4c, d). However, other molecules might also be involved in forming the complex, and other cells in addition to CD4^+^ T cells, such as macrophages and DCs, may also produce p19/CD5L in WT mice. The p19 and CD5L expression patterns were largely coordinated in various Th cells but not under certain Th-polarizing conditions, thus suggesting the production of p19 alone (Supplementary Fig. 2). This might be analogous to the results for p40, which is overproduced by activated macrophages and DCs, compared with the IL-12 heterodimer p35/p40^28^. Thus, this is the first report of the new p19 and CD5L heterodimer.

This study had some limitations, and several questions remain to be clarified in future studies. For example, do macrophages and DCs other than CD4^+^ T cells produce p19/CD5L? Do p19 and CD5L bind directly? Does a human homologue of mouse p19/CD5L exist and have similar functions? Does p19/CD5L affect the production of other cytokines and cells? What is the p19/CD5L receptor? What other signaling molecules are involved with p19/CD5L? Regarding the last questions, we further investigated the possibilities of involvement of IL-23R$\upalpha$ in the p19/CD5L singling and phosphorylation of the serine threonine protein kinase, AKT, and the mitogen-activated protein kinase, extracellular signal-regulated kinase (ERK), which are important for cell proliferation and survival^29^. We compared the proliferative activity of Ba/F3 cells expressing gp130/IL-12R$\upbeta$1/IL-12R$\upbeta$2 with and without IL-23R$\upalpha$ in response to hyper-p19/CD5L. Hyper-p19/CD5L induced proliferation of these Ba/F3 cells with IL-23R$\upalpha$ but not those without IL-23R$\upalpha$ (Supplementary Fig. 5), suggesting that IL-23R$\upalpha$ is possibly one of the receptor subunits for it. In response to hyper-p19/CD5L, AKT, but not ERK, was phosphorylated in the Ba/F3 cells expressing gp130/IL-12Rβ1/IL-12Rβ2/IL-23Rα (Supplementary Fig. 6). Recently, RNA sequencing data from human Th17 cells revealed the presence of p19 transcripts in the human sorted GM-CSF^+^IL-17A^+^CD4^+^ T cells^30^. Consistent with this report, we detected higher frequency of p19^+^CD5L^+^CD4^+^ T cells in human peripheral blood CD4^+^ T cells, which were activated under pathogenic Th17-polarizing conditions and consequently expressing higher frequency of GM-CSF^+^IL-17A^+^CD4^+^ T cells (Supplementary Fig. 7). Further studies are necessary to elucidate the signaling molecules of p19/CD5L and its role in human in more detail.

CD5L is a soluble protein member of the scavenger receptor cysteine-rich superfamily and plays various critical roles ranging from the modulation of leukocyte migration and inflammatory responses to the control of lipid metabolism^31,32^. Recently, Wang et al. demonstrated that CD5L is predominantly expressed in non-pathogenic Th17 cells and downregulated by IL-23 in pathogenic Th17 cells^11^; this is consistent with our results (Supplementary Fig. 2). CD5L was revealed to mediate this effect by modulating the intracellular lipidome, altering fatty acid composition and restricting cholesterol biosynthesis and resultant ligand availability for RORγt, the master transcription factor of Th17 cells^11^. Distinct from the intracellular role for CD5L in non-pathogenic Th17 cells, p19/CD5L appears to be secreted from various Th cells including Th0, ThGM, and pathogenic Th17 cells (Fig. 4d, Supplementary Fig. 2c,d), and it enhances the differentiation into GM-CSF-producing CD4^+^ T cells under various Th-polarizing conditions in an autocrine manner (Fig. 3, 6). Although Wang et al. showed increased susceptibility of CD5L-deficient mice to EAE^11^, our results seemed to indicate the opposite (Fig. 5a–d). This distinct susceptibility might be due to the different experimental conditions to induce EAE. Wang et al. used 40 μg of the MOG_35-55_ peptide for immunization, whereas we used 150 μg. However, when the amount of the MOG_35-55_ peptide was reduced from 150 µg to 40 µg for immunization, the EAE clinical score decreased in only the WT mice, and no significant difference was observed between the WT and CD5L-deficient mice (Supplementary Fig. 8). Moreover, Wang et al. used CD5L-deficient mice established by using ES cells, but we used the mice established by using CRISPR/Cas9, even though both mice were generated by Dr. T. Miyazaki’s group. CD5L and possibly p19/CD5L expressed in other cells such as macrophages and DCs may also affect susceptibility to EAE. Thus, the roles of CD5L and p19/CD5L on the induction of EAE seem to be opposite but are closely related, and therefore the delicate balance of abundance between them, that is, monomer versus heterodimer of CD5L, would determine the overall outcome in the susceptibility to EAE. Very recently, it was reported that the change of microbiota composition in the gastrointestinal tract is associated with the susceptibility to EAE^33,34^. Therefore, the possible differences of gut microbiota in mice between two facilities may also affect the different outcomes.

There are numerous cellular sources of GM-CSF including activated T cells and B cells, monocytes, macrophages, endothelial cells, and tumors, and among Th subsets, not only Th1, Th2, Th17 but also ThGM can produce GM-CSF^12,13^. The ThGM subset was demonstrated to be a distinct subset of Th cells generated by IL-2- or IL-7-activated STAT5 signaling and produce GM-CSF^14,15^. The GM-CSF then induces the production of IL-23 by macrophages and DCs, resulting in exacerbation of the CNS inflammation in EAE^14,15^. Among various Th subsets, p19/CD5L seems to most efficiently affect the generation of GM-CSF^+^CD4^+^T cells under Th0, ThGM-, and pathogenic Th17-polarizing conditions (Fig. 3, 6) possibly through activation of STAT5. Very recently, fate-mapping analysis of GM-CSF expression identified a discrete subset of inflammation-driving Th cells regulated by IL-23 and IL-1β^16^. Although specific ablation of this subset was demonstrated to unperturb tissue accumulation of other Th subsets such as Th1 and Th17, it strongly impaired the accumulation of tissue-invading phagocytes, which are the primary drivers of immunopathology in CNS inflammation^12^. It remains unknown whether these inflammation-driving Th cells are identical to the ThGM subset and what the relevance of p19/CD5L is to them.

Recently, a possible stand-alone function of p19 independent of p40 was demonstrated in that intracellular p19 can associate with gp130 in endothelial cells and augment the expression of adhesion molecules such as ICAM-1 and VCAM-1 with STAT3 activation^35^. p19 was thus implicated to contribute to inflammatory responses in giant-cell arteritis. Other than IL-23, another p19-related heterodimeric cytokine IL-39, which consists of p19 and EBI3, was also identified and shown to be secreted from activated B cells to activate neutrophils, resulting in inflammatory responses in lupus-like model mice^36,37^. IL-23 also plays a critical role for the activation and maintenance of Th17 cells in psoriasis, and the selective targeting of p19 rather than p40 has emerged as an effective treatment^38^. This is because IL-12p35- or IL-12Rβ2-deficient mice showed exacerbated psoriasis compared to WT mice, indicating that IL-12 protects from psoriasis skin inflammation^39^. Moreover, p19 targeting would also blockade not only IL-23 but also IL-39 and p19/CD5L, both of which might be involved in worsening psoriasis. Although further studies are necessary to prove it, these results would substantiate the concept that targeting of p19 is superior to targeting of p40 for the treatment of inflammatory and autoimmune diseases including psoriasis.

In conclusion, we have identified a new cytokine-like composite heterodimer of p19 and CD5L, and hope that it will be possibly termed as a new interleukin. This p19/CD5L contributes to the development of EAE by up-regulating GM-CSF. Thus, p19/CD5L could be a good target for treatment of autoimmune, inflammatory, and malignant diseases. In addition, p19/CD5L could be a good diagnostic marker for autoimmune and inflammatory diseases including multiple sclerosis and possibly psoriasis.

## Methods

### Mice

WT C57BL/6 mice and CD4-Cre-Tg mice were purchased from Sankyo Labo Service and Jackson Laboratory, respectively. p19^flox/flox^ mice (C57BL/6 background)^20^ and complete p19-deficient mice (C57BL/6 background)^19^ were kindly provided by Dr. P. Thakker and Dr. D. J. Cua, respectively. CD4-Cre-Tg mice and p19^flox/flox^ mice were mated to generate the CD4^+^ T-cell-specific conditional p19-deficient CD4-Cre/p19^flox/flox^ mice. CD5L-deficient mice were generated by using CRISPR/Cas9 method. All mice were maintained under pathogen-free conditions, and all animal experiments were approved by the President and by the Institutional Animal Care and Use Committee (IACUC) of Tokyo Medical University and performed in accordance with institutional, science community, and national guidelines for animal experimentation and the Animal Research: Reporting of In Vivo Experiments (ARRIVE) guidelines.

### Cell cultures

Mouse T cell hybridomas DO.BW (from Dr. T. Mizuochi), 2HCa2, 530, 447, KE8, 216, 118 (all from Dr. D. L. Woodland), 1D1-E7^40^ (from Dr. G. J. Thorbecke), 2B4^41^ (from Dr. T. Saito), T cell lymphoma DO11.10 (from Dr. H. Nariuchi), splenic CD4^+^ T cells, peritoneal exudate cell (PEC) macrophages, and CNS mononuclear cells were cultured at 37 °C under 5% CO_2_/95% air in RPMI1640 (Sigma-Aldrich) containing 10% fetal bovine serum (FBS), 50 μM 2-mercaptoethanol, and 100 μg/ml kanamycin (Meiji Seika). The mouse macrophage cell line, J744.1 (ATCC), and the human hepatocellular carcinoma cell line, HepG2 (from Dr. J. Miyazaki), were cultured in Dulbecco’s modified Eagle’s medium (DMEM) containing 10% FBS, and 100 U/ml penicillin and 100 μg/ml streptomycin (Invitrogen). IL-3-dependent mouse pro-B cell line Ba/F3 cells expressing gp130/IL-12Rβ1/IL-12Rβ2^42^ were kindly provided by Dr. J. Scheller. The Ba/F3 cells expressing gp130/IL-12Rβ1/IL-12Rβ2/IL-23Rα were then prepared via retroviral transduction of pMX-IL-23Rα-IRES-GFP into the Ba/F3 cells expressing gp130/IL-12Rβ1/IL-12Rβ2, then cultured in RPMI1640 containing 10% FBS, 50 μM 2-mercaptoethanol, and 100 μg/ml kanamycin in the presence of 1 ng/ml IL-23 (R&D Systems, Supplementary Fig. 3). HEK293T cells were cultured in DMEM (Invitrogen) containing 10% FBS, and 100 U/ml penicillin and 100 μg/ml streptomycin. The HEK293-F cell line is derived from HEK293 cells and was adapted to suspension culture in serum-free medium of FreeStyle 293 expression medium (Invitrogen).

### Plasmid

Mouse p19, p40, and EBI3 cDNAs were isolated by RT-PCR using total RNA prepared from concanavalin A-activated spleen cells, confirmed by sequencing, and subcloned into p3 × FLAG-CMV-14 vectors (Sigma-Aldrich), which has 3 × FLAG-epitope-tag sequence at C-terminal, or pCXN2^43^. pCMV3-CD5L-c-MYC was purchased from Sino Biological. According to the manufacturer’s information, the mouse CD5L nucleotide sequence (total 1,104 bp) is identical with the Gene Bank Reference ID NM_009690.2 except for the point mutations of 786A/G and 807 T/C, but these mutations does not cause amino acid variation. For preparation of hyper-p19/CD5L expression vector, the whole CD5L sequence, including signal sequence, followed by the flexible linker (Gly_4_Ser)_3_ linker, and then by a mature coding sequence of p19 were generated using standard PCR methods and cloned into p3 × FLAG-CMV-14 vector, which has 3 × FLAG-epitope-tag sequence at C-terminal. For preparation of hyper-p40/CD5L^21^ expression vector, the mature coding sequence of p40, followed by the flexible linker (Gly_4_Ser)_3_ linker, and then by the mature coding sequence of CD5L were generated by using standard PCR methods and cloned into p3 × FLAG-CMV-13 vector (Sigma-Aldrich), which has signal peptide sequence of preprotrypsin at N-terminal and 3 × FLAG-epitope-tag sequence at C-terminal. The p19 mutant, C55S (TGT → AGT), in which Ser replaced the intermolecular disulfide bond-forming cysteine, Cyc55, critical to binding p40, was generated by site-directed mutagenesis using recombinant PCR, confirmed by sequencing, and cloned into the p3 × FLAG-CMV-14 vector as described above.

### Recombinant protein

HEK293T cells cultured in DMEM containing 5% FBS were transiently transfected with the p3 × FLAG-CMV-14-hyper-p19/CD5L expression vector (20 μg) using FuGENE 6 (Promega). After 3 days, the culture supernatant (20 ml) was harvested, and hyper-p19/CD5L was purified via affinity chromatography with anti-p19 affinity gel, which was prepared using HiTRap NHS-activated HP column (GE Healthcare) and anti-p19 antibody (G23-8, Bio X Cell) per the manufacturer’s instructions (Supplementary Fig. 9a). Approximately 1 μg of purified protein was obtained, weighing ~ 70 kDa. Its purity was estimated to be > 95% as per SDS-PAGE analysis, followed by silver staining using Silver Stain MS Kit (Wako) and densitometrical analysis using Image Lab software (Bio-Rad, Supplementary Fig. 9b). The concentration was determined using CD5L as a standard in ELISA kit for CD5L (Sino Biological). Similarly, p19-3 × FLAG protein was prepared using anti-FLAG (M2) affinity gel (Sigma-Aldrich). Recombinant CD5L protein was purchased from R&D Systems.

### Induction and assessment of EAE

C57BL/6 mice were immunized by subcutaneous injection with 150 μg of MOG_35-55_ peptide (MEVGWYRSPFSRVVHLYRNGK, Sigma-Aldrich) emulsified in a complete Freund’s adjuvant containing 5 mg/ml H37RA (Difco Laboratories). On day 0 and 2 after immunization, 200 ng pertussis toxin (List Biological Laboratories) was given intravenously. Resultant mice were scored for clinical signs: 0, no clinical signs; 1, loss of tail tone; 2, hind limb weakness; 3, hind limb paralysis; 4, death.

### Histopathological evaluation

Mice were euthanized in a CO_2_ chamber and perfused through the left cardiac ventricle with cold phosphate-buffered saline (PBS). Spinal cords were then flushed out with PBS, and the brains were harvested. The tissues were then placed in 10% buffered formalin fixative overnight which was then changed to 75% ethanol. Tissues were then embedded in paraffin wax and cut into 4–6 μm sections on a vibrating microtome. Resultant sections were stained with standard hematoxylin and eosin (H&E) to observe cellular infiltration.

### Isolation of CNS mononuclear cells

Mice were euthanized in a CO_2_ chamber and then perfused through the left cardiac ventricle with cold PBS. Spinal cords were then flushed out with PBS, collected, and cut into small pieces; tissue was passed through a 40-μm cell strainer; and the remaining cellular content was separated from myelin debris by a 30%/70% Percoll gradient centrifugation. Mononuclear cells were removed from the interphase, washed, and resuspended in culture medium.

### Recall antigen response

Spleen cells (4 × 10^6^ cells/ml) and mononuclear cells (5 × 10^5^ cells/ml) infiltrating the CNS of immunized mice were restimulated with the MOG_35-55_ peptide (2, 5, and 20 μg/ml) for 3 days. Culture supernatant was then collected and analyzed via ELISA for GM-CSF, IFN-γ and IL-17A production.

### Th differentiation assay

Naive CD62L^+^CD4^+^ T cells were purified from spleen cells using the naive CD4^+^ T cell isolation kit and AutoMACS Pro (Miltenyi Biotec) (CD62L^+^CD4^+^ T cells > 95%). Naive CD4^+^ T cells (5 × 10^5^ cells/ml) were stimulated with plate-bound anti-CD3 (145-2C11, ATCC, 2–5 μg/ml) and anti-CD28 (37.51, BD Biosciences, 1–2 μg/ml) under Th1-polarizing conditions with IL-12 (Peprotech, 10 ng/ml) and anti-IL-4 (11B11, Bio $\times$ Cell), Th2-polarizing conditions with IL-4 (Peprotech, 20 ng/ml), anti-IFN-γ (XMG1.2, Bio $\times$ Cell), and anti-IL-12 (C17.8, from Drs. G. Trinchieri and H. Nariuchi), ThGM-polarizing conditions with IL-7 (Peprotech, 2 ng/ml), and anti-IFN-γ, non-pathogenic Th17-polarizing conditions with TGF-β1 (Peprotech, 5 ng/ml), hyper-IL-6^44^ (20 ng/ml, generated in house), anti-IL-4, and anti-IFN-γ, and pathogenic Th17-polarizing conditions with IL-1β (R&D Systems, 10 ng/ml), hyper-IL-6 (20 ng/ml), IL-23 (R&D Systems, 10 ng/ml), anti-IL-4, and anti-IFN-γ, Th conditions without any addition of cytokine and antibody, and Th0 conditions with only antibodies including anti-IFN-γ, anti-IL-12, and anti-IL-4. All anti-cytokine neutralizing monoclonal antibodies were used at 10 μg/ml. Unless indicated, the medium was exchanged on day 3 with fresh complete medium. On day 4, cells were collected, washed, and restimulated with 50 ng/ml phorbol 12-myristate 13-acetate (PMA, Sigma-Aldrich) and 500 ng/ml ionomycin (Sigma-Aldrich) in the presence of 5 μg/ml brefeldin A (BioLegend), followed by intracellular cytokine staining (described below).

### Flow cytometry

For intracellular cytokine staining, single-cell suspensions were restimulated for 4 h with 50 ng/ml PMA and 500 ng/ml ionomycin in the presence of 5 μg/ml brefeldin A. Cells were stained with Pacific blue-, FITC- or PE-conjugated anti-CD4 (GK1.5, BioLegend), fixed with Fixation Buffer (eBioscience) for 15 min, and permeabilized with Permeabilization Buffer (eBioscience) for 30 min. These cells were then stained intracellularly with FITC- or PerCP-Cy5.5-conjugated anti-IFN-γ (XMG1.2, BioLegend, eBioscience), APC-conjugated anti-IL-10 (JES5-16E3, BioLegend), PE-Cy7-conjugated anti-IL-17A (TC11-18H10, BioLegend), and PE-conjugated anti-GM-CSF (MP1-22E9, BioLegend). For intracellular staining of CD5L and p19, cells were stained with PE-conjugated anti-CD4 (GK1.5, BioLegend), fixed, permeabilized, and then stained intracellularly with biotin-conjugated anti-CD5L (R&D Systems), and eFluor 660-conjugated anti-p19 (fc23cpg, eBioscience), followed by Brilliant Violet 421-cojugated streptavidin (BioLegend). For intracellular staining of pY-STAT5, naive CD4^+^ T cells were stimulated with plate-bound anti-CD3 and anti-CD28 under Th conditions for 3 days, washed with PBS, and rested in 10% FBS medium for 6 h. These cells were then restimulated with recombinant p19, CD5L, p19/CD5L, and IL-2 (100 U/ml) for 20 min, and then fixed, permeabilized with ice-cold 90% methanol, and then stained intracellularly with Alexa Fluor 647-conjugated anti-pY-STAT5 (Tyr694) (C71E5, Cell Signaling) or Alexa Fluor 647-conjugated rabbit mAb IgG XP isotype control (DA1E, Cell Signaling) according to the manufacturers’ instructions. For cell surface expressions, following antibodies were used; gp130, IL-12Rβ1, IL-12Rβ2, IL-23Rα and WSX-1 were performed using PE-conjugated anti-gp130 (4H1B35, BioLegend), PE-conjugated anti-IL-12Rβ1 (R&D Systems), hamster anti-IL-12Rβ2 (HAM10B9, BD Biosciences) followed by PE-conjugated anti-armenian and syrian hamster IgG (BD Biosceinces), PE-conjugated anti-IL-23R (12B2B64, BioLegend) and rat anti-WSX-1 (clone 2918, kindly provided by Genentech) followed by PE-conjugated anti-rat IgG (Jackson ImmunoResearch). Resultant cells were analyzed on a FACSCanto II flow cytometer (BD Biosciences) followed by FlowJo Software v10 (Tree Star) (Supplementary Fig. 10). The compensation of FACS was routinely checked using fluorescent beads such as UltraComp eBeads Compensation Beads (invitrogen).

### RNA isolation and RT-PCR

Mouse T cell hybridomas and lymphoma and naive CD4^+^ T cells were stimulated with plate-bound anti-CD3 (2 μg/ml or indicated concentration) in the presence or absence of anti-CD28 (1 μg/ml) for 48 h or indicated time points. Naive CD4^+^ T cells (5 × 10^5^ cells/ml) were also stimulated with plate-bound anti-CD3 (2 μg/ml) and anti-CD28 (1 μg/ml) under various Th-polarizing conditions for 48 h. Total RNA was then extracted using a guanidine thiocyanate procedure or prepared using RNeasy Mini Kit (Qiagen), and cDNA was prepared using oligo(dT) primer and SuperScript III RT (Invitrogen). Semiquantitative PCR was performed using Taq DNA polymerase as previously described^45^. Cycle conditions were 94 °C for 40 s, 60 °C for 20 s, and 72 °C for 40 s. Hypoxanthine phosphoribosyl transferase (*HPRT*) was used as a housekeeping gene. Real-time quantitative PCR was performed using SYBR Premix Ex Taq II and a Thermal cycler Dice real-time system according to the manufacturer’s instructions (TAKARA). *HPRT* was used as a housekeeping gene to normalize mRNA. Relative expression of real-time PCR products was determined by using the ΔΔCt method to compare target gene and housekeeping gene mRNA expression. The specific primer pairs for each gene in semiquantitative RT-PCR analysis used were as follows: *p19*, 5′-AATAATGTGCCCCGTATCCA-3′ and 5′-GGCACTAAGGGCTCAGTCAG-3′; *p28*, 5′-TGAGGTTCAGGGCTATGTCC-3′ and 5′-AGGGGCAGCTTCTTTTCTTC-3′; *p35*, 5′-ACCTCAGTTTGGCCAGGGTC-3′ and 5′-CAAGGCACAGGGTCATCATC-3′; *p40*, 5′-ATGGCCATGTGGGAGCTGGAG-3′ and 5′-TTTGGTGCTTCACACTTCAGG-3′; *EBI3*, 5′-AACTCCACCAGATCCACGTC-3′ and 5′-GCGGAGTCGGTACTTGAGAG-3′; *IL-2*, 5′-TCCACTTCAAGCTCTACAG-3′ and 5′-GAGTCAAATCCAGAACATGCC-3′; *HPRT*, 5′-GTTGGATACAGGCCAGACTTTGTTG-3′ and 5′-GAGGGTAGGCTGGCCTATAGGCT-3′. The specific primer pairs for each gene in quantitative RT-PCR analysis used were as follows: *p19*, 5′- ACATGCACCAGCGGGACATA-3′ and 5′- CTTTGAAGATGTCAGAGTCAAGCAG-3′; *CD5L*, 5′- GTACCACGACTGTACCCACAAGGA-3′ and 5′- GAATGAGGGCCCACTGAACAA-3′; *GM-CSF*, 5′- AAGATATTCGAGCAGGGTCTACGG-3′ and 5′- CGCATAGGTGGTAACTTGTGTTTCA-3′; *HPRT*, 5′- TTGTTGTTGGATATGCCCTTGACTA-3′ and 5′- AGGCAGATGGCCACAGGACTA-3′.

### ELISA

Amount of p19 was determined by standard sandwich ELISA protocol using anti-p19 (5B2, eBioscience) as a capture antibody, biotin-conjugated anti-p19 (R&D Systems) as a detection antibody, and IL-23 as a standard protein for p19. The amount of p40 was determined by a standard sandwich ELISA protocol using anti-p40 (C15.6, BioLegend) as the capture antibody, biotin-conjugated anti-p40 (C17.8, BioLegend) as the detection antibody, and IL-12 (BioLegend) as the standard protein for p40. The amount of CD5L was determined by ELISA according to the manufacturers’ instructions (Sino Biological). The amount of p19/CD5L was determined by standard sandwich ELISA protocol using anti-p19 (5B2) as a capture antibody, biotin-conjugated anti-CD5L (R&D Systems) as a detection antibody, and the purified recombinant hyper-p19/CD5L as a standard protein. The concentration of the hyper-p19/CD5L was determined via CD5L-specific ELISA using CD5L as the standard protein. The amounts of IL-2, GM-CSF, IFN-γ, IL-17A and IL-10 in culture supernatants were determined by ELISA per the manufacturers’ instructions (R&D Systems or BD Biosciences). To detect p19/CD5L in the culture supernatants of activated CD4^+^ T cells, naive CD4^+^ T cells (5 × 10^5^ cells/ml) were stimulated with plate-bound anti-CD3 (5 μg/ml) and anti-CD28 (2 μg/ml) under Th, Th0 and pathogenic Th17-polarizing conditions for 3 days. The resultant culture supernatants were then concentrated by centrifugation using the Amicon Ultra-0.5-ml centrifugal filter with a 10-kDa Ultracel-10 membrane (Merck Millipore) and subjected to p19/CD5L-specific ELISA.

### Western blotting

Cells were then lysed at the indicated time points in a lysis buffer containing protease inhibitors, and the resultant cell lysates were separated by SDS-PAGE under reducing conditions and transferred to polyvinylidene difluoride membrane (Merck Millipore). The membrane was blocked, probed with antibodies against p19 (R&D Systems), EBI3 (Santa Cruz), CD5L (D-11 from Santa Cruz, GeneTex), STAT1 (Transduction Laboratories), STAT3 (Santa Cruz), STAT5 (Santa Cruz), AKT (Cell Signaling), ERK (Cell Signaling), pY-STAT1 (Cell Signaling), pY-STAT3 (Cell Signaling), pY-STAT5 (Cell Signaling), pAKT (Cell Signaling), pERK (Cell Signaling), or actin (Sigma-Aldrich), followed by an appropriate secondary antibody conjugated to horseradish peroxidase and visualized with an enhanced chemiluminescence detection system (GE Healthcare) per the manufacturer’s instructions. Immunoreactive bands were detected with a ChemiDoc XRS (Bio-Rad). To detect phosphorylation, naive CD4^+^ T cells (5 × 10^5^ cells/ml) were stimulated with plate-bound anti-CD3 (2 μg/ml) and anti-CD28 (1 μg/ml) under Th conditions for 3 days. The resultant cells were washed, rested in 10% FBS medium for 6 h and stimulated with various molecules for the indicated times, followed by western blotting with anti-pY-STATs, anti-pAKT and anti-pERK, and subsequently with antibodies against their total molecules.

### Immunoprecipitation

HEK293-F cells were transfected with p3 × FLAG-CMV-14-p19 and pCMV3-CD5L-c-MYC. Three days later, cell supernatants were collected, and cells were lysed in 1% Nonidet P-40 lysis buffer (10 mM Tris–HCl, pH 7.5, 150 mM NaCl, 1 mM EDTA) supplemented with protease inhibitor cocktail followed by centrifugation. The cell lysates or cell supernatants were then incubated with antibody (1 μg) against FLAG (M2, Sigma-Aldrich) or c-MYC (9E10, Santa Cruz) conjugated to protein G-Sepharose (GE Healthcare) for 2 h to overnight at 4 °C. After the beads were washed, the complexes were separated on an SDS-PAGE under reducing conditions and subjected to western blotting with biotin-conjugated anti-c-MYC (Santa Cruz) or biotin-conjugated anti-p19 (R&D Systems), respectively. Naive CD4^+^ T cells (5 × 10^5^ cells/ml) were stimulated with plate-bound anti-CD3 (5 μg/ml) and anti-CD28 (2 μg/ml) under pathogenic Th17-polarizing conditions for 3 days. The resultant culture supernatants were then concentrated by centrifugation using the Centriprep-10 K centrifugal filter with a 10-kDa Ultracel-10 membrane (Merck Millipore) and subjected to immunoprecipitation with anti-CD5L (D-11, Santa Cruz) followed by western blotting with biotin-conjugated anti-p19 (R&D Systems) as described above.

### Cell proliferation assay

Ba/F3 cells (2 × 10^4^ cells/ml) expressing gp130/IL-12Rβ1/IL-12Rβ2/IL-23Rα were serum-starved overnight, stimulated with various culture supernatants for 3 days, and pulsed with ^3^H-thymidine for the last 8 h. The ^3^H-thymidine incorporation into the DNA was measured using FilterMate cell harvester and TopCount microplate scintillation counter (PerkinElmer).

### Statistical analysis

Data are expressed as mean ± SD for each group. Statistical analyses were performed by using unpaired, two-tailed Student’s *t-*test for comparisons of two groups and one-way ANOVA with the Dunnett’s multiple comparison test for comparing more than three groups using GraphPad Prism v7 (GraphPad Software Inc.). *P* < 0.05 was considered to indicate a statistically significant difference.

## Supplementary Information

Supplementary information.

## Data Availability

All data generated or analyzed during this study are available from the corresponding author on reasonable request.
