# Author Correction: IL-23p19 and CD5 antigen-like form a possible novel heterodimeric cytokine and contribute to experimental autoimmune encephalomyelitis development

**DOI:** 10.1038/s41598-021-01575-x

**Published:** 2021-11-03

**Authors:** Hideaki Hasegawa, Izuru Mizoguchi, Naoko Orii, Shinya Inoue, Yasuhiro Katahira, Toshihiko Yoneto, Mingli Xu, Toru Miyazaki, Takayuki Yoshimoto

**Affiliations:** 1 Department of Immunoregulation, Institute of Medical Science, Tokyo Medical University, 6‑1‑1 Shinjuku, Shinjuku‑ku, Tokyo, 160‑8402 Japan; 2 Laboratory of Molecular Biomedicine for Pathogenesis, Center for Disease Biology and Integrative Medicine, Faculty of Medicine, The University of Tokyo, 7‑3‑1 Hongo, Bunkyo‑ku, Tokyo, 113‑0033 Japan

Correction to: *Scientific Reports* https://doi.org/10.1038/s41598-021-84624-9, published online 04 March 2021

The original version of this Article contained an error in Figure 6a where the graph numbers that calculate the average frequencies were incorrect. The original Figure 6 and accompanying legend appear below.

**Figure 6:**
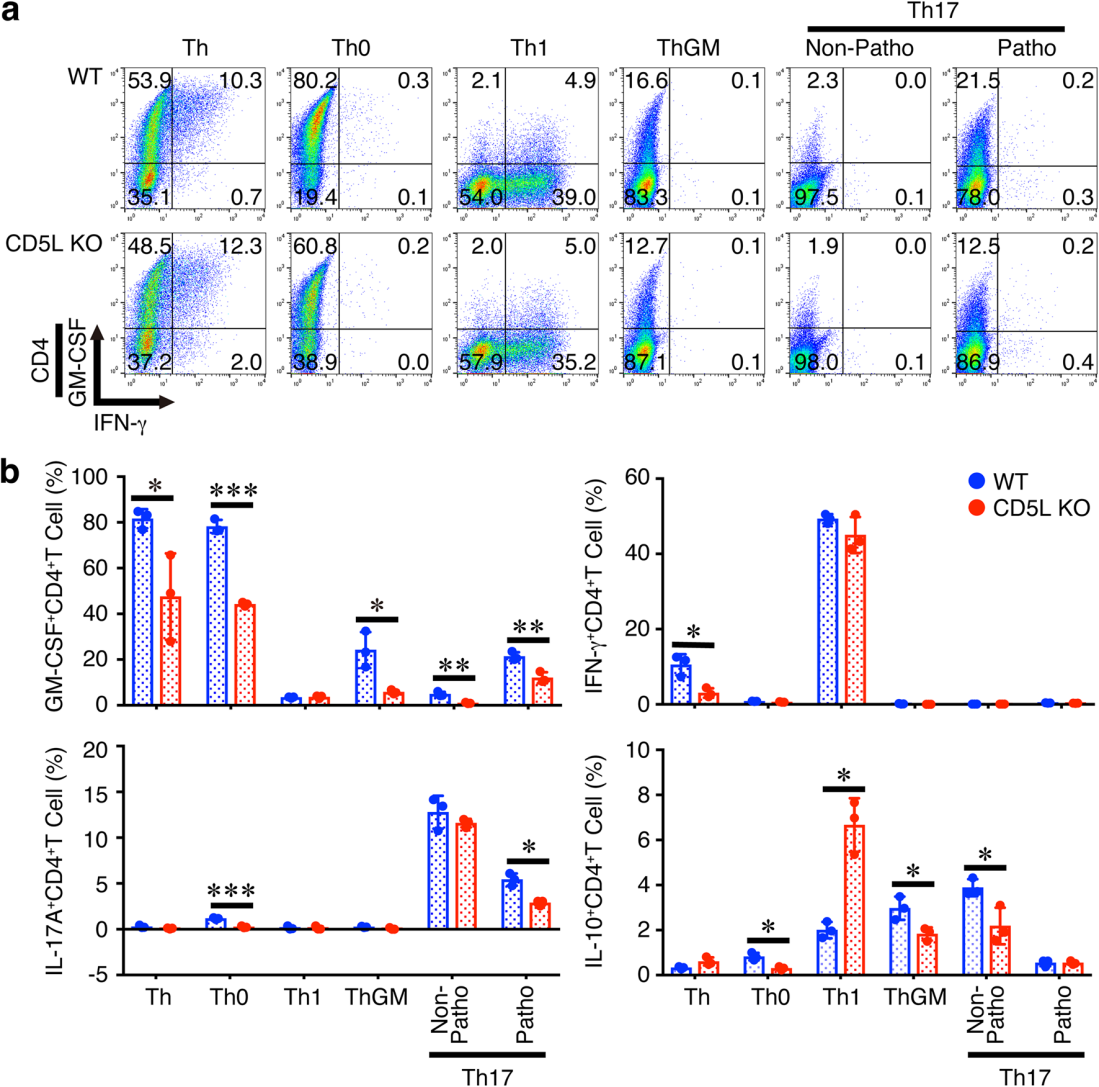
Differentiation into GM-CSF-producing CD4^+^ T cells is impaired in CD5L-deficient CD4^+^ T cells in vitro. Naive CD4^+^ T cells from WT mice or CD5L-deficient mice were stimulated with plate-coated anti-CD3 (2 μg/ml) and anti-CD28 (1 μg/ml) for 4 days under various Th-polarizing conditions; Th, Th0, Th1, ThGM, non-pathogenic Th17, and pathogenic Th17. These cells were then restimulated with PMA and ionomycin, and the intracellular cytokine staining was performed. Representative dot plots for GM-CSF, IL-17A, IFN-γ, and IL-10 in CD4^+^ T cells are shown (**a**), and average frequencies of respective CD4^+^ T cells were calculated and compared (**b**). Data are shown as mean ± SD (n = 3) and are representative of three independent experiments. *P* values were determined using unpaired, two-tailed Student’s *t*-test. *P < 0.05, **P < 0.01, ***P < 0.001.

The original Article has been corrected.

