# Supplementary material for: IL-23p19 and CD5 antigen-like form a possible novel heterodimeric cytokine and contribute to experimental autoimmune encephalomyelitis development
Source: Sci Rep. 2021 Mar 4;11:5266. doi: 10.1038/s41598-021-84624-9 (PMC7933155; doi:10.1038/s41598-021-84624-9)
Supplement: Supplementary file 1 — Supplementary information. [file 41598_2021_84624_MOESM1_ESM.pdf]

Supplementary information

IL-23p19 and CD5 antigen-like form a possible novel heterodimeric cytokine and contribute to experimental autoimmune encephalomyelitis development

Hideaki Hasegawa[1], Izuru Mizoguchi[1], Naoko Orii[1], Shinya Inoue[1], Yasuhiro Katahira[1], Toshihiko Yonteo[1], Mingli Xu[1], Toru Miyazaki[2], and Takayuki Yoshimoto[1*]

[1]Department of Immunoregulation, Institute of Medical Science, Tokyo Medical University, 6-1-1 Shinjuku, Shinjuku-ku, Tokyo 160-8402, Japan

[2]Laboratory of Molecular Biomedicine for Pathogenesis, Center for Disease Biology and Integrative Medicine, Faculty of Medicine, The University of Tokyo, 7-3-1 Hongo, Bunkyo-ku, Tokyo 113-0033, Japan

*Correspondence should be addressed to Takayuki Yoshimoto, Department of Immunoregulation, Institute of Medical Science, Tokyo Medical University, 6-1-1 Shinjuku, Shinjuku-ku, Tokyo 160-8402, Japan. E-mail: yoshimot@tokyo-med.ac.jp

Supplementary Methods

Human naive CD4$^+$ T cell preparation. Fresh human peripheral blood was collected from healthy volunteers and mononuclear cells were immediately purified by using Lympholyte-H (Cedarlane) density gradient centrifugation. Naive CD4$^+$ T cells were further purified from peripheral blood mononuclear cells by negative selection using the naive CD4$^+$ T cell isolation kit and AutoMACS Pro (Miltenyi Biotec). The purity was analyzed by flow cytometry and routinely more than 95%. This study was approved by the institutional review board of Tokyo Medical University (no. 3339). Written informed consent was obtained from all participants in accordance with the Declaration of Helsinki.

Th differentiation assay. Human naive CD4$^+$ T cells were stimulated with anti-CD3 and anti-CD28 using Dynabeads Human T-Activator CD3/CD28 (ThermoFisher) under Th1-polarizing conditions with human IL-12 (kindly provided from Dr. Gately, 10 ng/ml) and anti-human IL-4 (MP4-25D2, BioLegend, 10 μg/ml), Th2-polarizing conditions with human IL-4 (BioLegend, 20 ng/ml) and anti-human IFN-γ (AF-285-NA, R&D, 5 μg/ml), and Th17-polarizing conditions with human IL-1β (Peprotech, 10 ng/ml), human IL-6 (BioLegend, 20 ng/ml), human IL-23 (eBioscience, 10 ng/ml), anti-human IFN-γ, anti-human IL-4, and Th conditions without any addition of cytokine and antibody.

Flow cytometry. For intracellular human cytokine staining, single-cell suspensions were restimulated for 4 h with 50 ng/ml PMA and 500 ng/ml ionomycin in the presence of 5 μg/ml brefeldin A. Cells were stained with FICT-conjugated anti-human CD4 (OKT4, BioLegend), fixed with Fixation Buffer (eBioscience) for 15 min, and permeabilized with Permeabilization Buffer (eBioscience) for 30 min. These cells were then stained intracellularly with PE-conjugated anti-human p19 (727753, R&D), APC-conjugated anti-human CD5L (CD5L/2951, Novusbio), Pacific blue-conjugated anti-human GM-

CSF (BVD2-21C11, BioLegend), and PE-Cy7-conjugated anti-human IL-17A (eBio64DE17, eBioscience).

Supplementary Table 1. Comparison of EAE susceptibility between control $p19^{flox/flox}$ mice and $CD4^{+}$ T-cell-specific conditional p19-deficient (CD4-Cre/$p19^{flox/flox}$) mice.

Mice	Incidence Rate	Day of Onset	Maximum Score
$p19^{flox/flox}$	7 / 7	15.3 ± 3.5	3.0 ± 0.9
CD4-Cre/$p19^{flox/flox}$	6 / 7	18.6 ± 4.6	1.3 ± 0.7
P value		$P = 0.259$	$P < 0.05$

Supplementary Table 2. Comparison of EAE susceptibility between control WT mice and CD5L-deficient mice.

Mice	Incidence Rate	Day of Onset	Maximum Score
WT	6 / 6	9.8 ± 2.3	3.2 ± 0.4
CD5L-deficient	5 / 6	14.6 ± 4.3	1.0 ± 0.6
P value		$P = 0.061$	$P < 0.001$

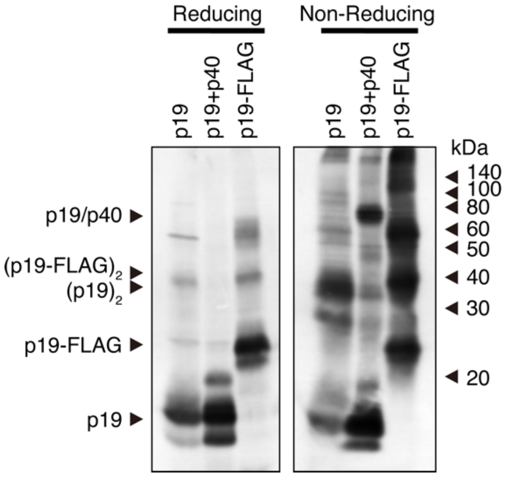

Supplementary Figure 1. Possible formation of a p19 homodimer in the culture supernatants of cells overexpressed with the p19 expression vector. HEK293T cells were transfected with expression vectors of p3×FLAG-CMV-14-p19 without a FLAG tag or p3×FLAG-CMV-14-p19 alone and cotransfected with expression vectors of p3×FLAG-CMV-14-p19 without a FLAG tag or p3×FLAG-CMV-14-p40 without a FLAG tag. After 3 days, the culture supernatants were subjected to western blotting using anti-p19 under reducing and nonreducing conditions. Data are representative of two independent experiments.

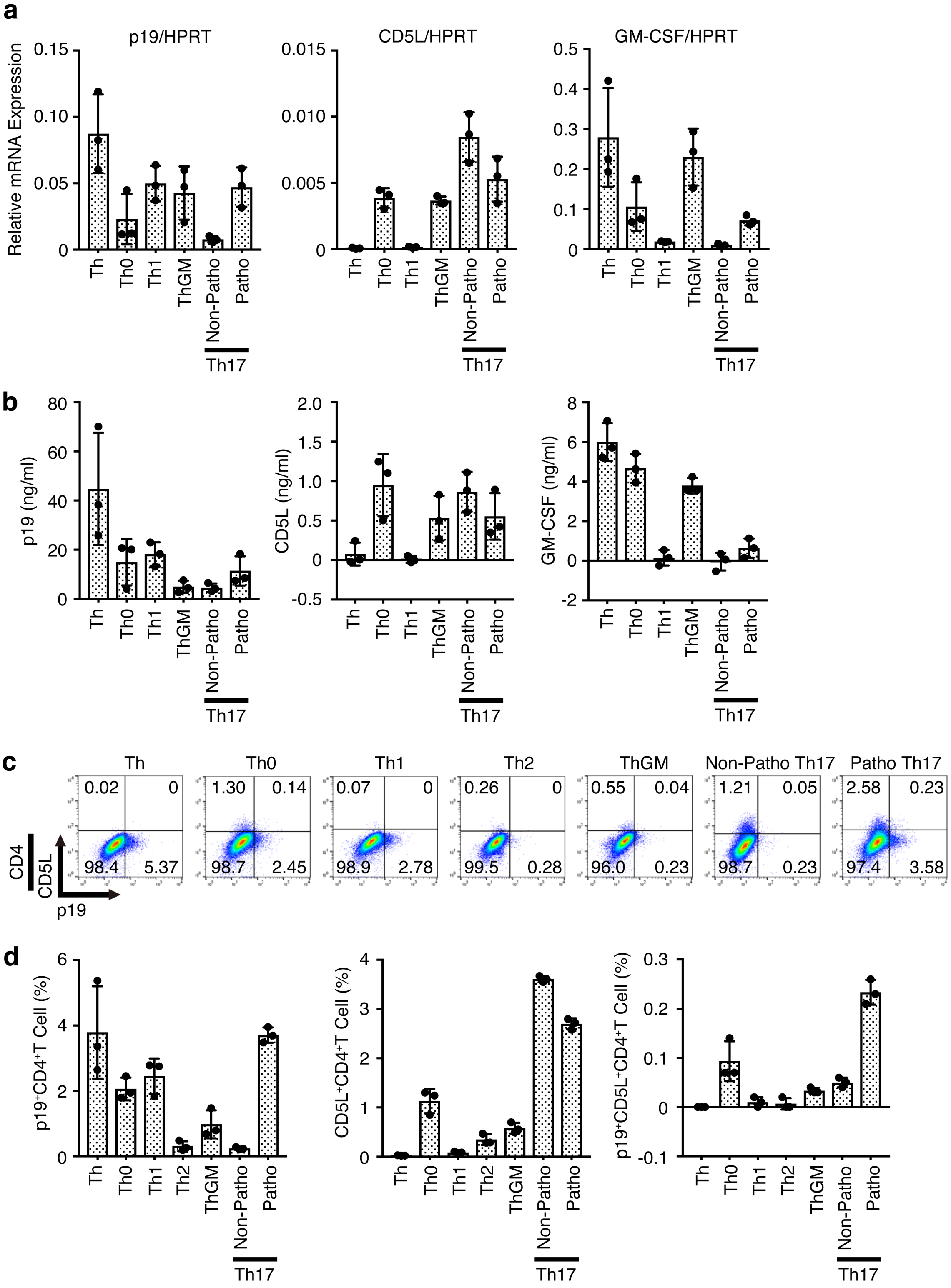

Supplementary Figure 2. Correlation of p19, CD5L, p19/CD5L, and GM-CSF under various Th-polarizing conditions. **a**, **b** Naive CD4^{+} T cells from WT mice were stimulated with plate-coated anti-CD3 (2 μg/ml) and anti-CD28 (1 μg/ml) for 2 days under various Th-polarizing conditions; Th, Th0, Th1, ThGM, non-pathogenic Th17, and pathogenic

Th17. Total RNA was extracted and subjected to quantitative RT-PCR analysis (**a**). After 3 days later, culture supernatants were collected and analyzed for protein expression of p19 and CD5L by ELISA (**b**). Note that a recombinant single-chain IL-23 (10 ng/ml), which can be detected by p19-specific ELISA, was included in the culture medium under pathogenic Th17-polarizing conditions. **c**, **d** Naive $CD4^+$ T cells were also stimulated with plate-coated anti-CD3 (5 μg/ml) and anti-CD28 (2 μg/ml) under various Th-polarizing conditions for 2 days, then intracellularly stained for p19 and CD5L after restimulation with PMA and ionomycin. Representative dot plots were shown (**c**). Data are shown as the mean ± SD (n = 3) and are representative of at least three independent experiments.

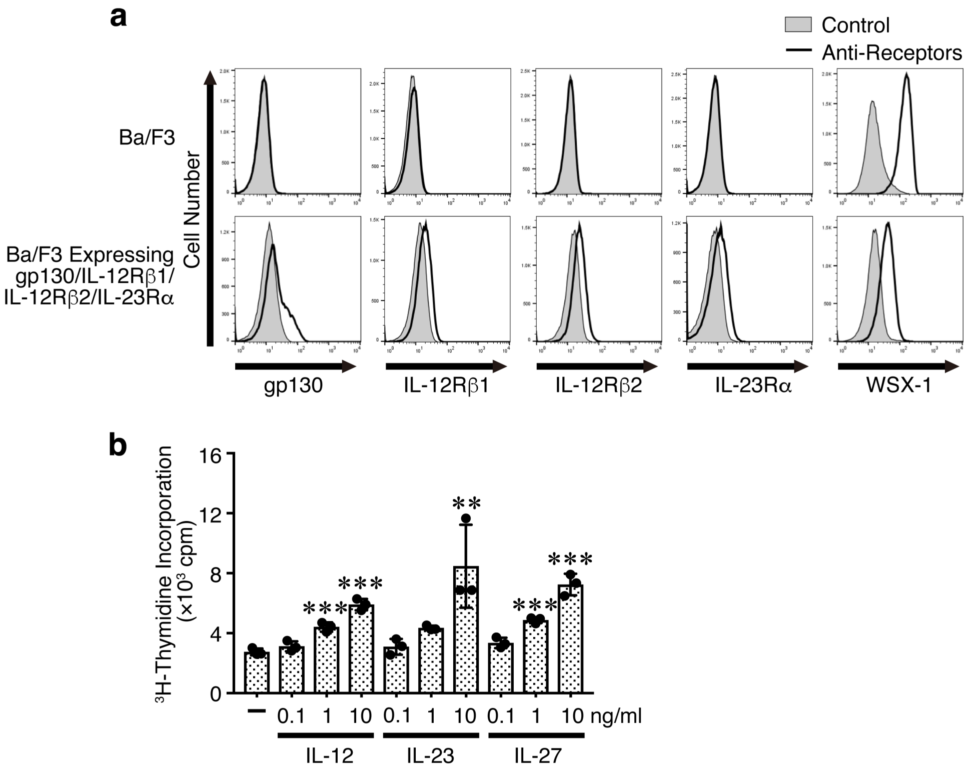

Supplementary Figure 3. Characterization of Ba/F3 cells expressing gp130/IL-12Rβ1/IL-12Rβ2/IL-23Rα. Cell surface expressions of gp130, IL-12Rβ1, IL-12Rβ2, IL-23Rα and WSX-1 on parental Ba/F3 and Ba/F3 cells transfected with expression vectors for gp130, IL-12Rβ1, IL-12Rβ2 and IL-23Rα were analyzed via FACS (**a**). Proliferative activity of these cells in response to IL-12, IL-23 and IL-27 was determined (**b**). Data are shown as the mean ± SD in triplicate and are representative of three independent experiments. *P* values were determined using one-way ANOVA. $^{**}P < 0.01$, $^{***}P < 0.001$.

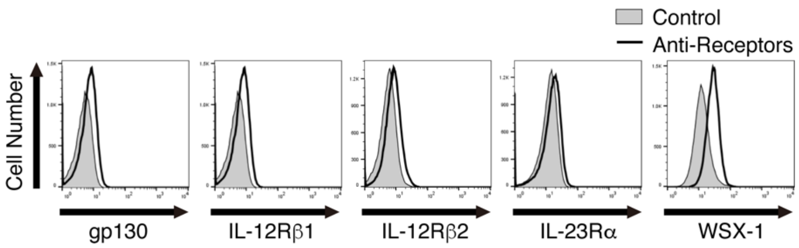

Supplementary Figure 4. Cell surface expression of activated CD4$^+$ T cells. Naive CD4$^+$ T cells from WT mice were stimulated with plate-coated anti-CD3 (2 μg/ml) and anti-CD28 (1 μg/ml) under Th conditions for 3 days, and their cell surface expressions of gp130, IL-12Rβ1, IL-12Rβ2, IL-23Rα and WSX-1 were analyzed via FACS. Data are representative of two independent experiments.

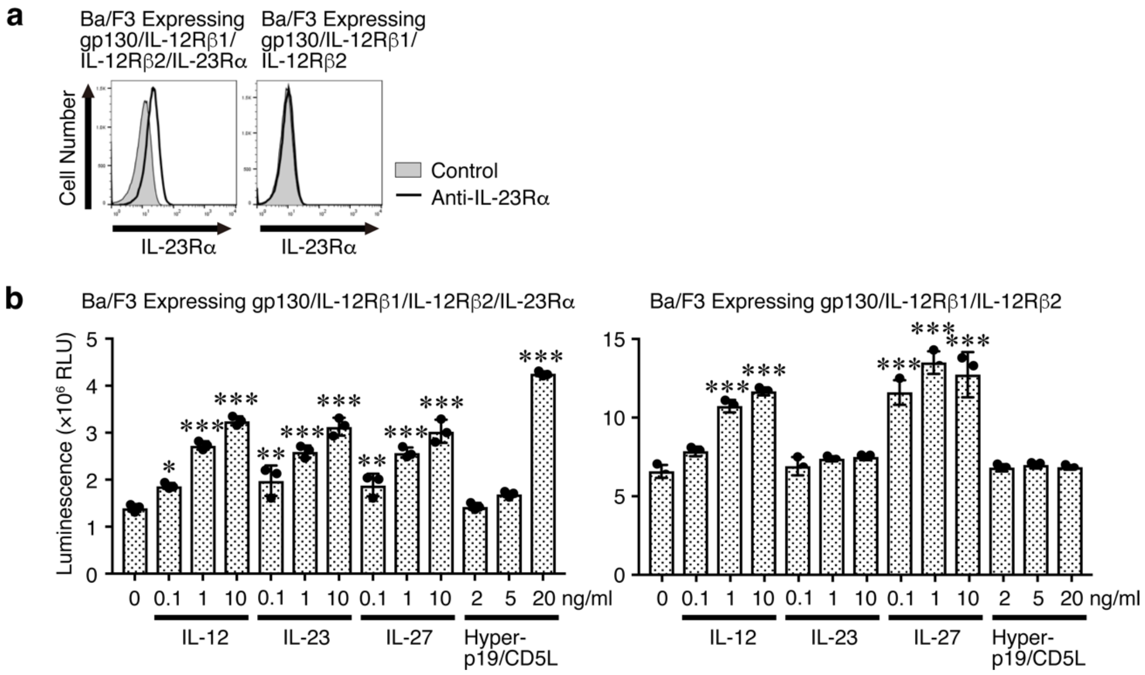

Supplementary Figure 5. IL-23Rα is possibly one of the receptor subunits for p19/CD5L.Ba/F3 cells (1 × 10^4 cells/200 μl) expressing gp130/IL-12Rβ1/IL-12Rβ2/IL-23Rα and Ba/F3 cells (3 × 10^3 cells/200 μl) expressing gp130/IL-12Rβ1/IL-12Rβ2 were analyzed for their cell surface expression of IL-23Rα (**a**) and their proliferative activities in response to IL-12, IL-23, IL-27, and hyper-p19/CD5L were determined after 3 days using CellTiter-Glo 2.0 Viability Assay kit (Promega) (**b**). Data are shown as the mean ± SD in triplicate and are representative of three independent experiments. *P* values were determined using one-way ANOVA. *$P < 0.05$, **$P < 0.01$, ***$P < 0.001$.

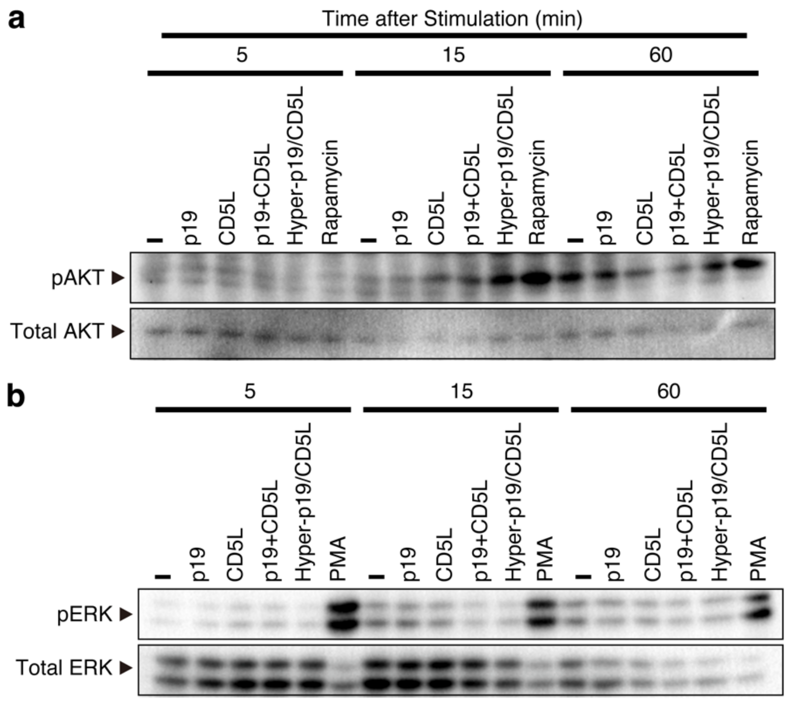

Supplementary Figure 6. Hyper-p19/CD5L induced phosphorylation of AKT but not ERK. Ba/F3 cells expressing gp130/IL-12Rβ1/IL-12Rβ2/IL-23Rα were unstimulated (−) or stimulated with purified recombinant p19, CD5L, a mixture of p19 and CD5L, hyper-p19/CD5L (all 20 ng/ml), and rapamycin (Sigma-Aldrich, positive control for AKT phosphorylation, 5 μM) (**a**) or PMA (positive control for ERK phosphorylation, 5 μM) (**b**) for 5, 15 and 60 min, then subjected to western blotting with anti-pAKT or anti-pERK and subsequently anti-total AKT or anti-total ERK. Data are representative of two independent experiments.

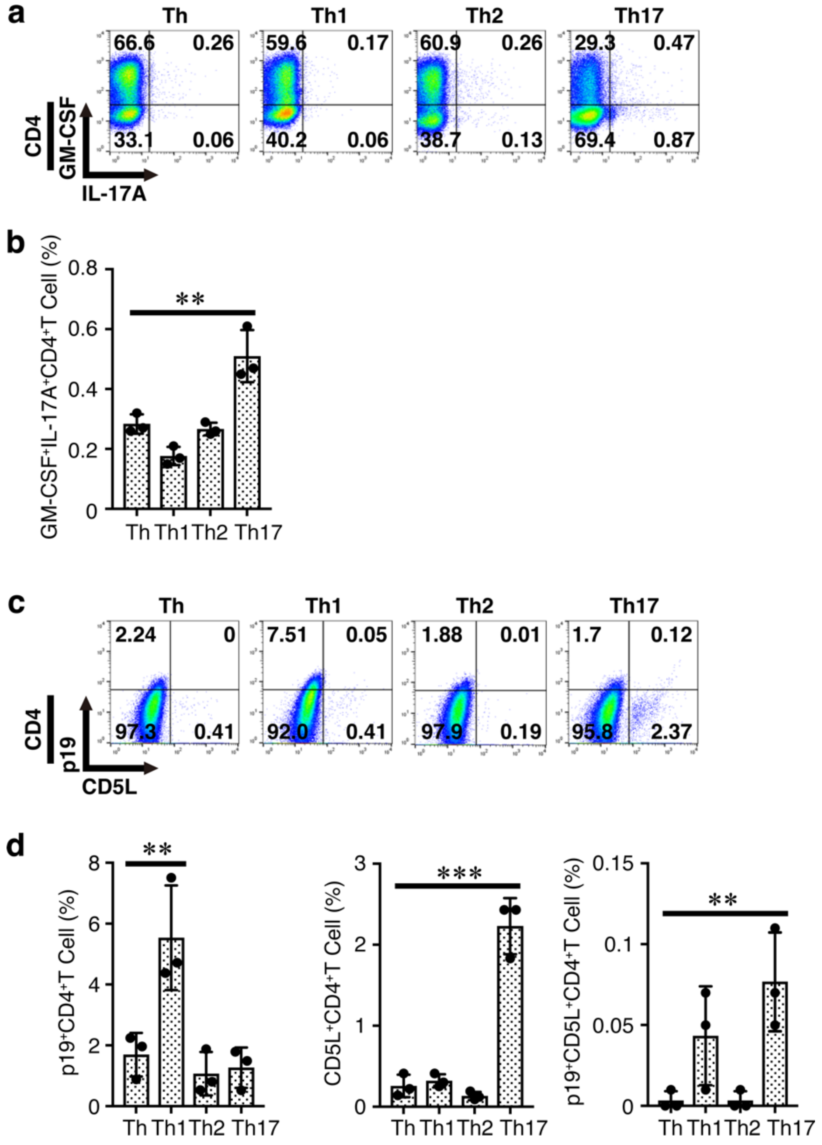

Supplementary Figure 7. Higher frequency of p19$^+$CD5L$^+$CD4$^+$ T cells in human CD4$^+$ T cells activated under pathogenic Th17-polarizing conditions and expressing higher frequency of GM-CSF$^+$IL-17A$^+$CD4$^+$ T cells. Human peripheral blood-derived naive CD4$^+$ T cells were stimulated under Th1-, Th2-, and Th17-polarizing conditions together with Th conditions for 6 days with IL-2, and then intracellularly stained for GM-CSF, IL-17A (**a**, **b**), p19, and CD5L (**c**, **d**) after restimulation with PMA and ionomycin. Data are shown as the mean ± SD in triplicate and are representative of two independent experiments. *P* values were determined using one-way ANOVA. $^{**}P < 0.01$, $^{***}P < 0.001$.

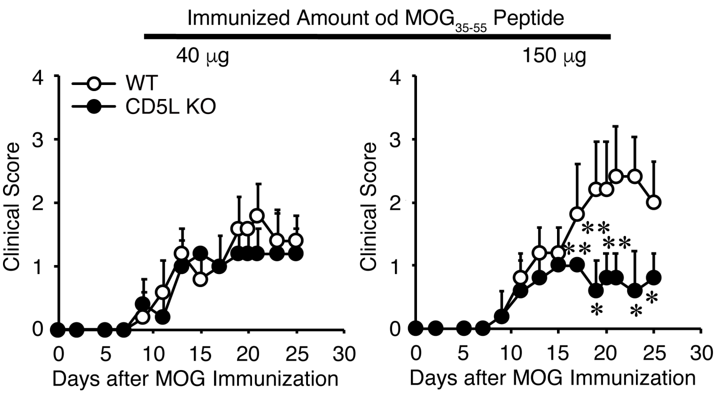

Supplementary Figure 8. Effect of dose of MOG_{35-55} peptide used for immunization on the susceptibility of WT and CD5L-deficient mice to develop EAE. WT mice or CD5L-deficient mice were immunized with the MOG_{35-55} peptide 40 μg or 150 μg, and their clinical scores were monitored over time. Data are shown as the mean ± SD (n = 5) and representative of two independent experiments. *P* values were determined using unpaired two-tailed Student's *t*-tests. $^{*}P < 0.05$.

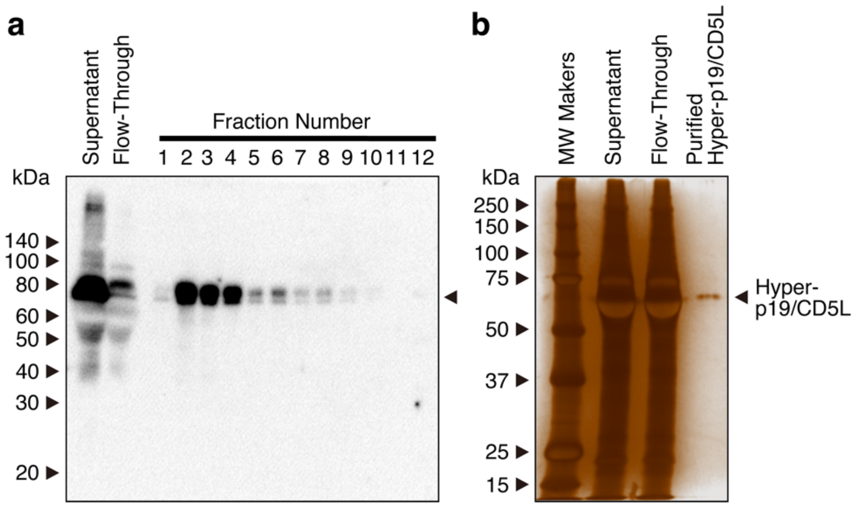

Supplementary Figure 9. Characterization of purification procedure by western blotting with anti-CD5L and silver staining of recombinant hyper-p19/CD5L protein. HEK293T cells were transiently transfected with the p3×FLAG-CMV-14-hyper-p19/CD5L expression vector. After 3 days, the culture supernatant was harvested, and hyper-p19/CD5L was purified by affinity chromatography with an anti-p19 affinity column. Each fraction eluted from the affinity column, the culture supernatant before applying the column, and the flow-through were subjected to western blotting analysis with anti-CD5L (Santa Cruz) (**a**). The purity was determined by silver staining following SDS-PAGE and estimated to be >95% via densitometrical analysis using Image Lab software (**b**).

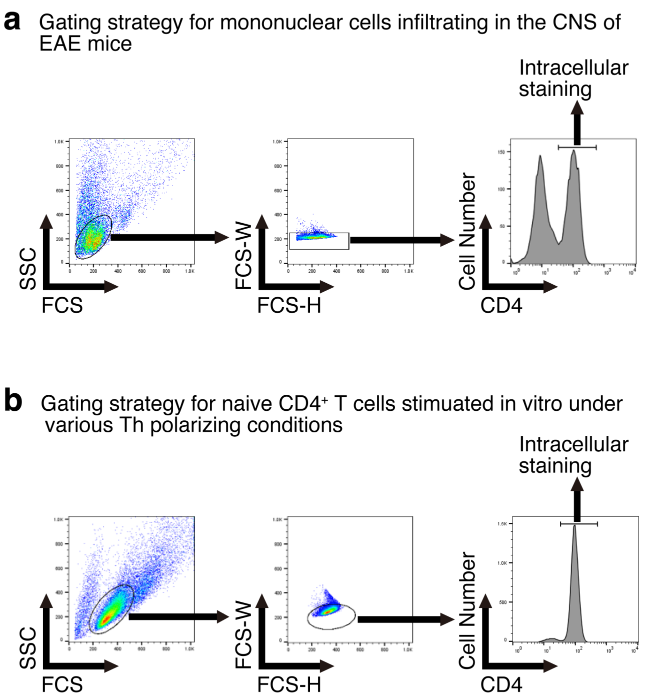

Supplementary Figure 10. Gating strategy with flow cytometry. Gating strategy for mononuclear cells infiltrating in the CNS of EAE mice (**a**) and for naive $CD4^+$ T cells stimulated in vitro under various Th-polarizing conditions (**b**).
